#  Performance evaluation of ARIMA, VAR, ANN, GRU, and LSTM for multivariate healthcare time-series prediction: a rigorous, reproducible benchmarking study with ablation analysis on MIMIC-III and eICU

**DOI:** 10.3389/frai.2026.1873285

**Published:** 2026-09-24

**Authors:** Md. Tazul Islam, Muhammad Hanif, Samuel Kim

**Affiliations:** 1 Department of Applied Mathematics, Noakhali Science and Technology University, Noakhali, Choumohoni, Bangladesh; 2 Department of Computer Science, Stanford University, Stanford, CA, United States

**Keywords:** ARIMA, clinical alarm simulation, eICU, GRU, ICU time-series forecasting, LSTM, MIMIC-III, VAR

## Abstract

**Introduction:**

Accurate forecasting of physiological time-series in the intensive care unit (ICU) is critical for early-warning systems, deterioration detection, and dynamic resource allocation. Yet the comparative literature suffers from five persistent methodological deficiencies: non-unified experimental conditions, heterogeneous preprocessing, univariate classical baselines compared against multivariate neural models, single-run point estimates without variance, and underspecified prediction tasks.

**Methods:**

This paper addresses all five deficiencies through a standardized, reproducible benchmarking study comparing Autoregressive Integrated Moving Average (ARIMA), Vector Autoregression (VAR), Artificial Neural Networks (ANN), Gated Recurrent Units (GRU), and Long Short-Term Memory (LSTM) networks on one-step-ahead (1-h-ahead) prediction of heart rate, respiratory rate, systolic and diastolic blood pressure, and oxygen saturation across two large-scale ICU cohorts (8,000 MIMIC-III and 15,000 eICU admissions). VAR is introduced as a multivariate linear baseline alongside univariate ARIMA, enabling a three-way decomposition of performance gains into multivariate modeling, nonlinear approximation, and gated temporal memory. All experiments are repeated over 10 independent seeds and reported as mean ± 95% confidence interval with Diebold-Mariano, paired *t*-test, and Bonferroni-corrected ANOVA significance testing. Seven categories of ablation study, a clinical alarm simulation, a leave-one-hospital-out protocol, and a subgroup fairness analysis are additionally conducted.

**Results:**

LSTM achieves the highest performance on MIMIC-III (RMSE 0.082 ± 0.004, *R*^2^ 0.940 ± 0.008, directional accuracy 90.1%±0.9%), with GRU reaching near-equivalent accuracy at 64% of training cost. The layered decomposition reveals that gated temporal memory provides the largest marginal gain (−47% to −53% RMSE over ANN), followed by nonlinear approximation (−19% over VAR) and multivariate modeling (−13% over ARIMA). Ablation identifies normalization as the single most critical preprocessing step (+79.3% RMSE without it) and a window length of *l* = 10 as optimal. The clinical alarm simulation demonstrates a 38.4% false alarm rate reduction for LSTM relative to threshold-only monitoring, and the leave-one-hospital-out experiment confirms cross-institutional generalizability with consistent model ranking.

**Discussion:**

Subgroup fairness analysis identifies a performance gap for severely ill patients (Sequential Organ Failure Assessment score ≥ 8, ratio 1.27) warranting targeted future work. These results establish a reusable, methodologically rigorous benchmark for ICU physiological forecasting and provide actionable model-selection guidance for clinical deployment.

## 1 Introduction

### 1.1 Clinical motivation

Physiological deterioration in the ICU is rarely sudden; it is typically preceded by subtle, progressive changes in vital signs that can be detected hours before a critical event (Andersen et al., 2020; Yoon et al., 2016). Heart rate acceleration, progressive hypoxaemia, widening pulse pressure, or tachypnoeic trends are individually weak signals but collectively carry strong predictive information when modeled over time. Contemporary ICU monitoring systems generate continuous time-series at resolutions of seconds to minutes, producing datasets of extraordinary richness that remain largely untapped for prospective prediction (Pollard et al., 2018; Johnson et al., 2016). The gap between the data richness of modern ICUs and the statistical sophistication of most clinical decision-support tools represents a major opportunity for machine learning research. Recent work on predictive modeling for pandemic forecasting has further underscored the importance of reliable time-series forecasting frameworks in healthcare settings (Baker et al., 2025b).

The clinical cost of this gap is concrete. Threshold-based alarm systems, which fire when a single vital sign crosses a fixed threshold, generate false alarm rates exceeding 85% in many ICU environments (Jones et al., 2023). The resulting alarm fatigue desensitizes clinicians, delays responses to genuine deterioration, and increases both cognitive load and patient stress. Predictive models capable of forecasting physiological trajectories could substantially reduce false alarms while improving sensitivity to genuine deterioration, but only if those models are accurate, reliable, and generalizable across patient populations.

### 1.2 The benchmarking problem

Despite extensive literature on time-series forecasting methods in healthcare, the field lacks a methodological consensus on how to compare models fairly. We identify five recurring deficiencies in existing comparative studies:

1. **Non-unified experimental conditions**. Models are trained and tested under different preprocessing protocols, data splits, and hardware configurations, making direct comparison unreliable (Alsheheri, 2025; Wang et al., 2019).
2. **Heterogeneous preprocessing**. Differences in imputation strategy, resampling interval, and normalization scheme can produce performance differences as large as those attributable to model architecture (Singh et al., 2023).
3. **Biased baseline selection**. ARIMA is consistently used in *univariate* mode while being compared against *multivariate* deep learning models. This confounds the contribution of multivariate modeling capacity with that of architectural sophistication, systematically overstating the advantage of neural approaches (Zhou et al., 2024).
4. **Single-run point estimates**. Reporting a single training run's results without variance estimates or confidence intervals is insufficient to distinguish genuine performance differences from random initialization effects, especially when differences are small (Kumar et al., 2022).
5. **Underspecified prediction tasks**. Many studies describe the goal as “forecasting physiological signals” without specifying the prediction horizon, target variables, or clinical use case, limiting the clinical interpretability of findings.

This paper corrects all five deficiencies simultaneously within a unified experimental framework. Additionally, it introduces two evaluation dimensions absent from all prior comparative ICU forecasting studies to our knowledge: (i) a clinical alarm simulation that translates model accuracy into operationally meaningful false-alarm and sensitivity metrics, and (ii) a subgroup fairness analysis stratifying performance by age, sex, and illness severity.

### 1.3 Contributions

This paper makes the following specific contributions:

First, the prediction objective is formally specified as one-step-ahead (1-h-ahead) forecasting of five ICU physiological variables, enabling unambiguous replication. Second, VAR is introduced as a multivariate linear baseline alongside univariate ARIMA, enabling a three-way decomposition of performance gains into contributions from multivariate linear modeling, nonlinear function approximation, and gated temporal memory. Third, GRU is evaluated alongside LSTM to determine whether the full LSTM gating complexity is necessary, with both accuracy and computational cost benchmarked. Fourth, all metrics are reported over 10 independent runs as mean ± 95% CI, with full significance testing including the Diebold-Mariano test with Newey-West correction. Fifth, seven categories of ablation study quantify the contributions of preprocessing, sequence length, depth, unit count, dropout, learning rate schedule, and input signal selection. Sixth, a leave-one-hospital-out protocol on the multi-center eICU dataset quantifies generalization to unseen institutions. Seventh, model predictions are post-processed through a trajectory-based alarm rule to estimate false alarm rate and sensitivity, bridging the gap between forecasting accuracy and clinical utility. Eighth, performance is stratified by patient age, sex, and SOFA score to assess whether accuracy disparities exist across clinically important subgroups. Finally, all preprocessing scripts, model implementations, training configurations, and random seeds are fully documented and released for reproducibility.

### 1.4 Paper organization

Section 2 reviews related work across model families and evaluation practices. Section 3 formalizes the prediction problem. Section 4 describes all model architectures. Section 5 details datasets, cohort definition, and preprocessing. Section 6 defines the full evaluation framework. Section 7 presents all experimental results. Section 8 interprets findings, addresses limitations, and outlines future directions. Section 9 concludes.

## 2 Related work

### 2.1 Classical statistical models

ARIMA, introduced by Box and Jenkins (Box and Jenkins, 1970), remains one of the most widely used forecasting methods in healthcare due to its interpretability, low computational cost, and well-understood theoretical properties (Alsheheri, 2025). Healthcare applications include prediction of ICU census and bed demand (Zhao et al., 2023), hospital admission rates, and short-horizon physiological trends. ARIMA's core limitation, namely its assumption of linearity and stationarity, is particularly problematic for ICU physiological signals, which exhibit highly nonlinear dynamics, sudden state transitions (e.g., at clinical interventions), and heteroscedastic noise (Hasan et al., 2024).

ARIMAX extends ARIMA with exogenous inputs, and SARIMA captures seasonal components; both have seen limited uptake in ICU applications. Vector Autoregression (VAR) (Box and Jenkins, 1970) jointly models multiple time series, capturing linear cross-variable dependencies that univariate ARIMA misses. Despite its theoretical suitability as a multivariate linear baseline, VAR is conspicuously absent from nearly all prior ICU forecasting benchmarks, representing a methodological gap that directly affects the fairness of classical-vs-neural comparisons.

### 2.2 Feedforward neural networks

Feedforward ANNs can approximate arbitrary continuous functions (universal approximation theorem), making them effective for nonlinear regression tasks (Dickie, 2007). In healthcare forecasting, ANNs have been applied to patient risk scoring, in-hospital mortality prediction, and disease progression modeling (Singh et al., 2023). However, standard feedforward architectures treat each time window as an independent observation; they have no intrinsic mechanism for propagating information across windows beyond the input features explicitly provided. When applied to sequential ICU data, this architectural limitation means that long-range temporal dependencies, such as a gradual multi-hour trend toward hypoxaemia, can only be captured if they are explicitly encoded in the input window, which requires careful feature engineering and imposes hard limits on the temporal horizon accessible to the model.

### 2.3 Recurrent neural networks and gated architectures

Recurrent Neural Networks (RNNs) process sequences step-by-step, maintaining a hidden state that accumulates temporal context (Hochreiter and Schmidhuber, 1997). Vanilla RNNs are limited by vanishing and exploding gradient problems that emerge during backpropagation through time, preventing the learning of dependencies longer than approximately 10–15 time steps. LSTM networks (Hochreiter and Schmidhuber, 1997) overcome this through gated memory cells with three learned control gates (input, forget, output), enabling selective retention of information across arbitrarily long sequences. GRU (Cho et al., 2014) achieves comparable expressivity with two gates (update, reset) and approximately 25% fewer parameters than LSTM.

Multiple recent studies have demonstrated that LSTM and GRU outperform ARIMA and ANN on ICU prediction tasks: mortality prediction on MIMIC-III (Kumar et al., 2024; Yu et al., 2019), length-of-stay estimation (Hasan et al., 2024; Jiang et al., 2022), ICU readmission (Lim et al., 2025; Xue et al., 2018), and deterioration detection (Eini-Porat et al., 2022; Alghatani et al., 2022). However, these studies differ in preprocessing, cohort definition, baseline models, and evaluation metrics, making direct synthesis difficult and precluding confident conclusions about the magnitude of performance advantages.

### 2.4 Transformer-based models and foundation architectures

Transformer models (Lim et al., 2021) replace recurrence with multi-head self-attention, enabling parallelizable training and direct modeling of arbitrary-distance dependencies. Temporal Fusion Transformers (TFT) (Lim et al., 2021) have set state-of-the-art results on multiple long-horizon forecasting benchmarks. Autoformer, Informer, and PatchTST are further specialized for time-series. In clinical AI, large foundation models pre-trained on EHR data (e.g., Clinical BERT, MedBERT) and time-series pre-training strategies (e.g., masked autoencoding of physiological sequences) represent an emerging frontier (Yadav et al., 2025). These architectures are not included in this benchmark as our focus is on the classical-to-LSTM comparison space, but they represent the principal direction for future work.

### 2.5 Hybrid and ensemble methods

Hybrid ARIMA-LSTM models leverage ARIMA for the linear component of a time series and LSTM for the residual nonlinear component, improving over either alone (Li et al., 2024). Prophet-LSTM hybrids for COVID-19 ICU demand (Borges et al., 2022) and wavelet-LSTM combinations for length-of-stay prediction (Jiang et al., 2022) have demonstrated consistent improvements. Stacked ensemble methods combining diverse model classes offer robustness to individual model failure modes (Iwase et al., 2021). While promising, hybrid methods introduce additional complexity in training and hyperparameter optimization, and their components are not independently interpretable in the same way as single-model baselines. Collaborative frameworks for disease prediction using multiple machine learning models have also shown promise in multi-model integration for clinical applications (Baker et al., 2025a).

### 2.6 Evaluation practice and fairness in clinical AI

A growing literature in clinical AI highlights that model evaluation must go beyond aggregate accuracy metrics. Subgroup performance disparities across age, sex, race, or illness severity can mask the fact that a model performs well on average but poorly for specific patient groups (Eini-Porat et al., 2022). Similarly, the translation of forecasting accuracy to clinical utility requires simulation of operational scenarios (e.g., alarm systems, escalation triggers) rather than relying solely on error metrics that clinicians cannot directly interpret (Jones et al., 2023). This paper incorporates both subgroup fairness analysis and alarm simulation, filling a gap in the prior ICU forecasting evaluation literature.

### 2.7 Summary of research gap

Table 1 summarizes the key methodological features of representative prior comparative studies and this paper.

**Table 1:** Comparison of methodological features across representative prior comparative ICU forecasting studies.

References	VAR	GRU	CI	Ablation	2+ Datasets	Subgroup	Alarm sim.	Code
Zhou et al. (2024)	×	×	×	×	×	×	×	×
Wang et al. (2019)	×	×	×	×	×	×	×	×
Kumar et al. (2022)	×	✓	×	×	×	×	×	×
Alsheheri (2025)	×	✓	×	×	×	×	×	×
Alghatani et al. (2022)	×	×	×	×	✓	×	×	×
**This paper**	✓	✓	✓	✓	✓	✓	✓	✓

✓, included; ×, absent.

## 3 Problem formulation

### 3.1 Notation

Let ${\text{X}}_{t}\in {\mathbb{R}}^{d}$ denote the vector of *d* = 5 physiological observations at hourly time step *t*, comprising ${\left[{\text{HR}}_{t},{\text{RR}}_{t},{\text{BP}}_{t}^{\text{sys}},{\text{BP}}_{t}^{\text{dia}},{\text{SpO}}_{2,t}\right]}^{⊤}$. Let T={1,2,…,T} denote the full observation timeline for a given ICU admission. A multivariate time-series window of length *l* ending at time *t* is denoted ${\text{W}}_{t}=\left({\text{X}}_{t-l+1},\ldots ,{\text{X}}_{t}\right)\in {\mathbb{R}}^{l\times d}$.

### 3.2 One-step-ahead forecasting objective

The prediction objective is formally defined as learning a function $f_{\theta }:{\mathbb{R}}^{l\times d}\to {\mathbb{R}}^{d}$ that maps a look-back window **W**_*t*_ to a prediction of the next observation, as given in Equation 1:


$$\begin{array}{l} {\hat{\text{X}}}_{t+1}=f_{\theta }\left({\text{W}}_{t}\right)=f_{\theta }\left({\text{X}}_{t-l+1},{\text{X}}_{t-l+2},\ldots ,{\text{X}}_{t}\right) \end{array}$$ (1)

Parameters **θ** are optimized by minimizing mean squared error over the training set, as defined in Equation 2:


$$\begin{array}{l} \theta^{*}=\arg\underset{\theta }{\min}\frac{1}{\left|D_{\text{train}}\right|}\underset{\left(t,{\text{X}}_{t+1}\right)\in D_{\text{train}}}{\sum }\|{\text{X}}_{t+1}-f_{\theta }\left({\text{W}}_{t}\right){\|}_{2}^{2} \end{array}$$ (2)

### 3.3 Scope and assumptions

This work focuses on one-step-ahead (1-h-ahead) prediction. This horizon is chosen because: (i) it is the shortest actionable horizon for bedside clinical response; (ii) it enables the sharpest separation of model capabilities; and (iii) it avoids the compounding error accumulation of multi-step autoregressive generation, which would confound architectural comparisons. Univariate prediction (per-variable) is used for ARIMA; all other models predict all *d* = 5 variables jointly. The study does not address multi-step forecasting, event detection (e.g., mortality, sepsis onset), or treatment recommendation; these are considered out of scope.

## 4 Methodology

### 4.1 Unified experimental pipeline

All models share the following pipeline:


$$\begin{array}{c} Raw\;ICU\;Admissions\to Cohort\;Selection\to Preprocessing\to \\ Chronological\;Split\to Model\;Training\to One-Step-Ahead\\ Inference\to Evaluation \end{array}$$

Identical preprocessing parameters, data splits, and evaluation code are applied across all model classes, ensuring that observed performance differences are attributable solely to model architecture. Figure 1 provides an end-to-end visual summary of the five stages (data sources, preprocessing, model family, training/inference, and evaluation) that constitute this unified pipeline.

**Figure 1:**
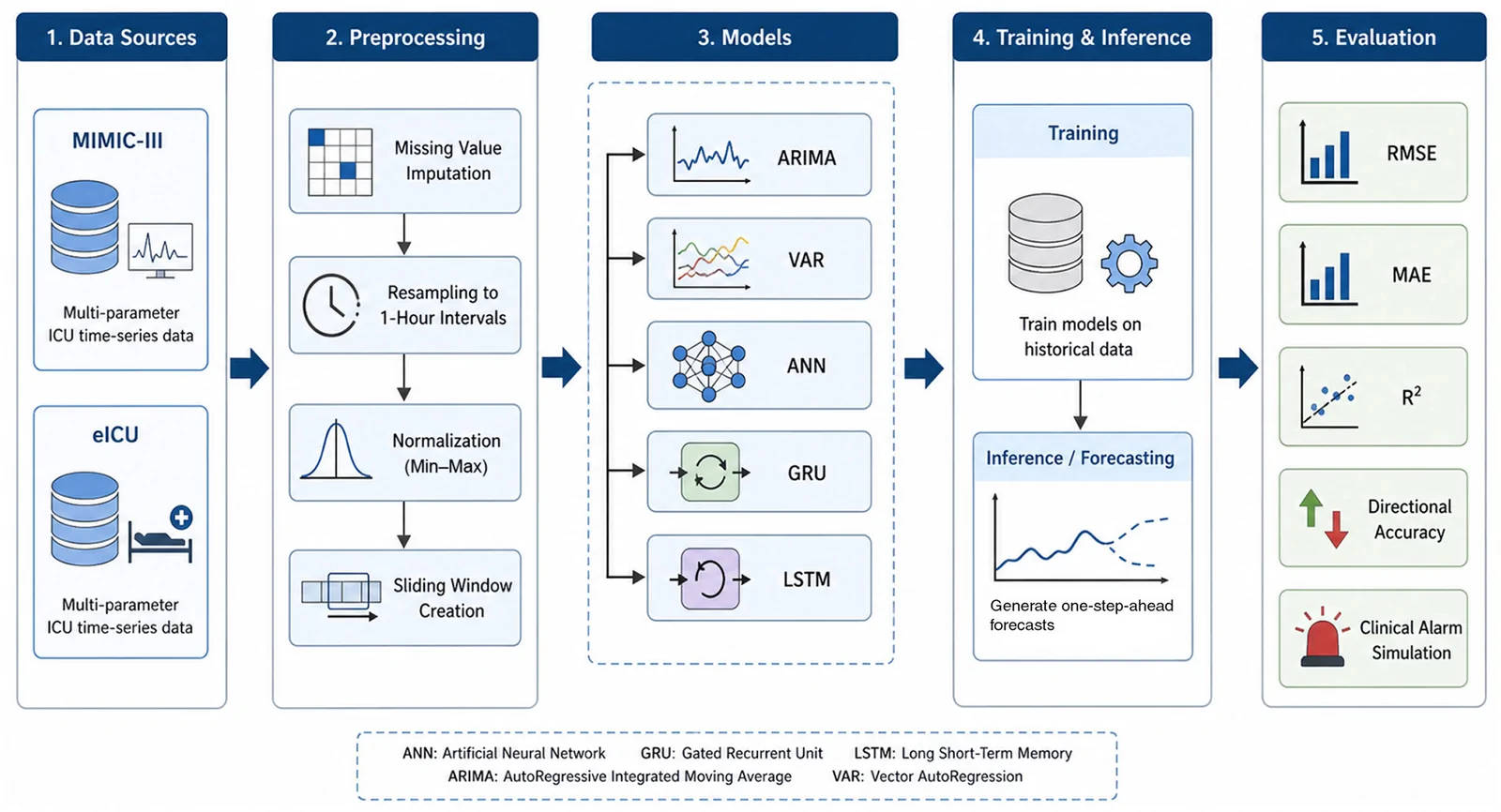
Unified experimental pipeline for the benchmarking study. Raw multi-parameter ICU records from MIMIC-III and eICU pass through a shared preprocessing module (missing-value imputation, resampling to 1-h intervals, Min -Max normalization, and sliding-window construction) before being supplied to all five candidate models (ARIMA, VAR, ANN, GRU, and LSTM) under identical training and inference conditions. Predictions are evaluated with RMSE, MAE, *R*^2^, directional accuracy, and a clinical alarm simulation, ensuring that observed performance differences are attributable solely to model architecture.

### 4.2 ARIMA

ARIMA(*p, d, q*) (Box and Jenkins, 1970) models a univariate time series as shown in Equation 3:


$$\begin{array}{l} \phi \left(B\right){\left(1-B\right)}^{d}y_{t}=\mu +\theta \left(B\right)\varepsilon_{t},\;\varepsilon_{t}\overset{\text{iid}}{\sim }N\left(0,\sigma^{2}\right) \end{array}$$ (3)

where *B* is the backshift operator, $\phi \left(B\right)=1-\overset{p}{\underset{i=1}{\sum }}\phi_{i}B^{i}$ and $\theta \left(B\right)=1+\overset{q}{\underset{j=1}{\sum }}\theta_{j}B^{j}$ are the AR and MA polynomials, and *d* is the differencing order. The one-step-ahead forecast is given by Equation 4:


$$\begin{array}{l} {ŷ}_{t+1|t}=\mu +\overset{p}{\underset{i=1}{\sum }}\phi_{i}y_{t-i+1}+\overset{q}{\underset{j=1}{\sum }}\theta_{j}\varepsilon_{t-j+1} \end{array}$$ (4)

Orders (*p, d, q*) are selected per variable via Akaike Information Criterion (AIC) grid search over *p, q*∈{0, 1, 2, 3} and *d*∈{0, 1, 2}, subject to stationarity verified by the Augmented Dickey-Fuller (ADF) test (α = 0.05). Each of the *d* = 5 physiological variables is modeled as an independent univariate ARIMA process; the *d*-dimensional prediction is formed by concatenating the five univariate forecasts.

### 4.3 Vector Autoregression (VAR)

VAR(*p*) (Box and Jenkins, 1970) jointly models all *d* variables as shown in Equation 5:


$$\begin{array}{l} {\text{X}}_{t}=\nu +\overset{p}{\underset{k=1}{\sum }}{\text{A}}_{k}{\text{X}}_{t-k}+\varepsilon_{t},\;\varepsilon_{t}\sim N\left(0,\Sigma \right) \end{array}$$ (5)

where ${\text{A}}_{k}\in {\mathbb{R}}^{d\times d}$ are lag-*k* coefficient matrices estimated by Ordinary Least Squares (OLS), and **Σ** is the contemporaneous covariance matrix. Lag order *p* is selected by minimizing the AIC over *p*∈{1, …, 10}. VAR is the natural multivariate linear baseline; its inclusion allows us to decompose the performance advantage of neural models into (a) multivariate linear modeling and (b) nonlinear/temporal components separately.

### 4.4 Artificial Neural Network (ANN)

Let ${\text{h}}^{\left(0\right)}=\text{vec}\left({\text{W}}_{t}\right)\in {\mathbb{R}}^{l\cdot d}$ denote the flattened input window. A two-hidden-layer feedforward network is defined by Equations 6, 7:


$$\begin{array}{l} {\text{h}}^{\left(k\right)}=\text{ReLU}\left({\text{W}}^{\left(k\right)}{\text{Drop}}_{p_{d}}\left({\text{h}}^{\left(k-1\right)}\right)+{\text{b}}^{\left(k\right)}\right),\;k=1,2 \end{array}$$ (6)

$$\begin{array}{l} {\hat{\text{X}}}_{t+1}={\text{W}}^{\left(3\right)}{\text{h}}^{\left(2\right)}+{\text{b}}^{\left(3\right)} \end{array}$$ (7)

where Drop_*p*_*d*__(·) applies dropout with rate *p*_*d*_ = 0.2 during training. Each hidden layer has 64 neurons. This architecture captures nonlinear input-output mappings but lacks any recurrent mechanism; temporal context is provided entirely through the flattened window **h**^(0)^, as illustrated in Figure 2.

**Figure 2:**
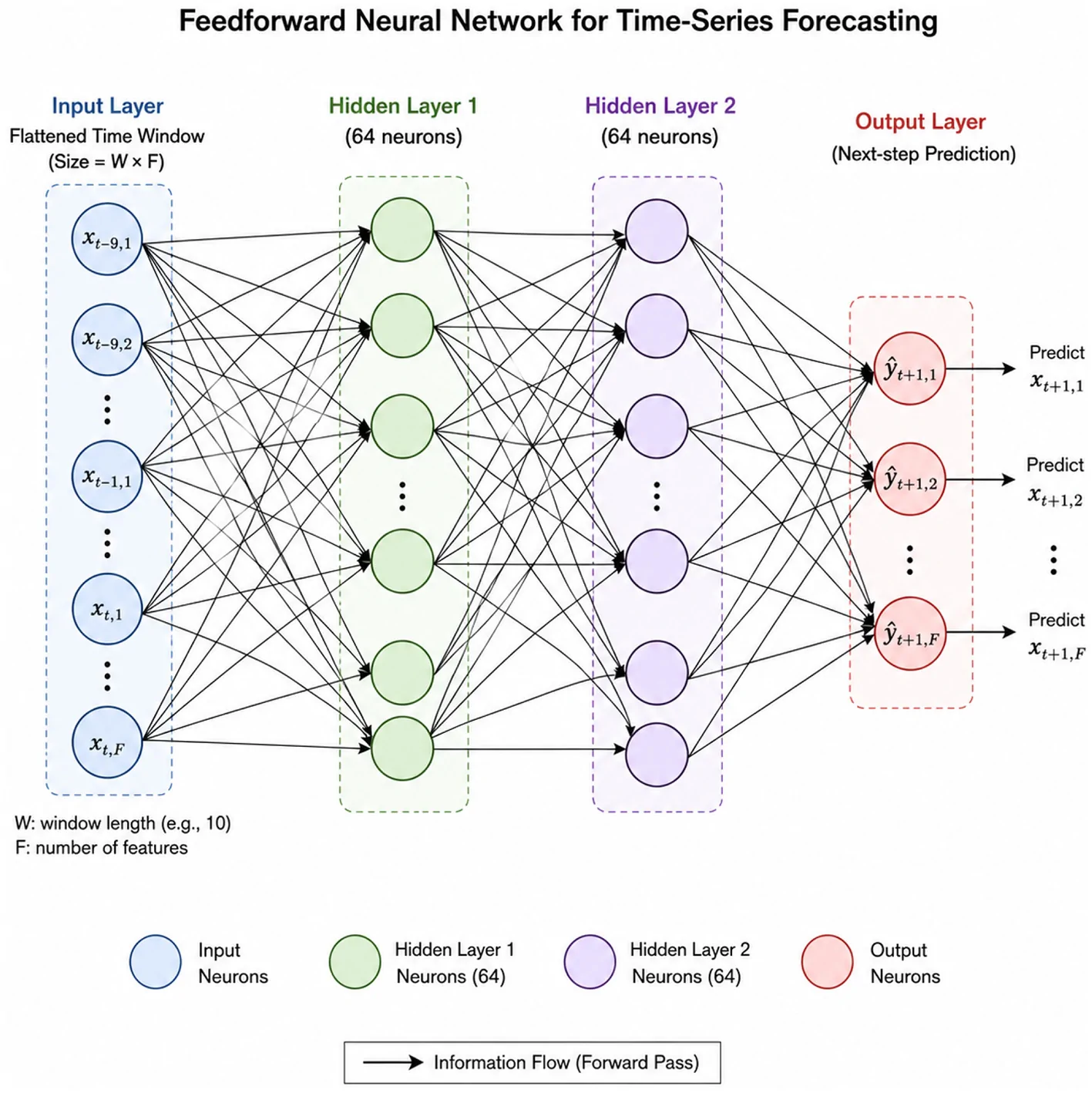
Feedforward artificial neural network (ANN) architecture used as a nonlinear, non-recurrent baseline. The flattened time window of size *W* × *F* (window length *W* = 10, *F* = 5 physiological features) is fed through two fully-connected hidden layers of 64 neurons each (ReLU activation, dropout *p*_*d*_ = 0.2). A linear output layer then produces the joint next-step prediction ${\hat{\text{X}}}_{t+1}$ for all five physiological variables. Temporal context is encoded only through the flattened input window; no recurrent state is propagated across windows.

### 4.5 Gated Recurrent Unit (GRU)

GRU (Cho et al., 2014) processes the input sequence (**X**_*t*−*l*+1_, …, **X**_*t*_) step-by-step according to Equations 8–11:


$$\begin{array}{l} {\text{z}}_{\tau }=\sigma \left({\text{W}}_{z}\left[{\text{h}}_{\tau -1};{\text{x}}_{\tau }\right]+{\text{b}}_{z}\right)\;(\text{update gate}) \end{array}$$ (8)

$$\begin{array}{l} {\text{r}}_{\tau }=\sigma \left({\text{W}}_{r}\left[{\text{h}}_{\tau -1};{\text{x}}_{\tau }\right]+{\text{b}}_{r}\right)\;(\text{reset gate}) \end{array}$$ (9)

$$\begin{array}{l} {\tilde{\text{h}}}_{\tau }=\tanh\left({\text{W}}_{h}\left[{\text{r}}_{\tau }⊙{\text{h}}_{\tau -1};{\text{x}}_{\tau }\right]+{\text{b}}_{h}\right)\;(\text{candidate state}) \end{array}$$ (10)

$$\begin{array}{l} {\text{h}}_{\tau }=\left(1-{\text{z}}_{\tau }\right)⊙{\text{h}}_{\tau -1}+{\text{z}}_{\tau }⊙{\tilde{\text{h}}}_{\tau }\;(\text{output state}) \end{array}$$ (11)

The final hidden state **h**_*t*_ is passed through a linear layer to produce ${\hat{\text{X}}}_{t+1}$. The GRU architecture uses one recurrent layer (64 units), dropout (*p*_*d*_ = 0.2), and a dense output layer. The unrolled recurrence and gate-level computations of a single GRU cell are depicted in Figure 3.

**Figure 3:**
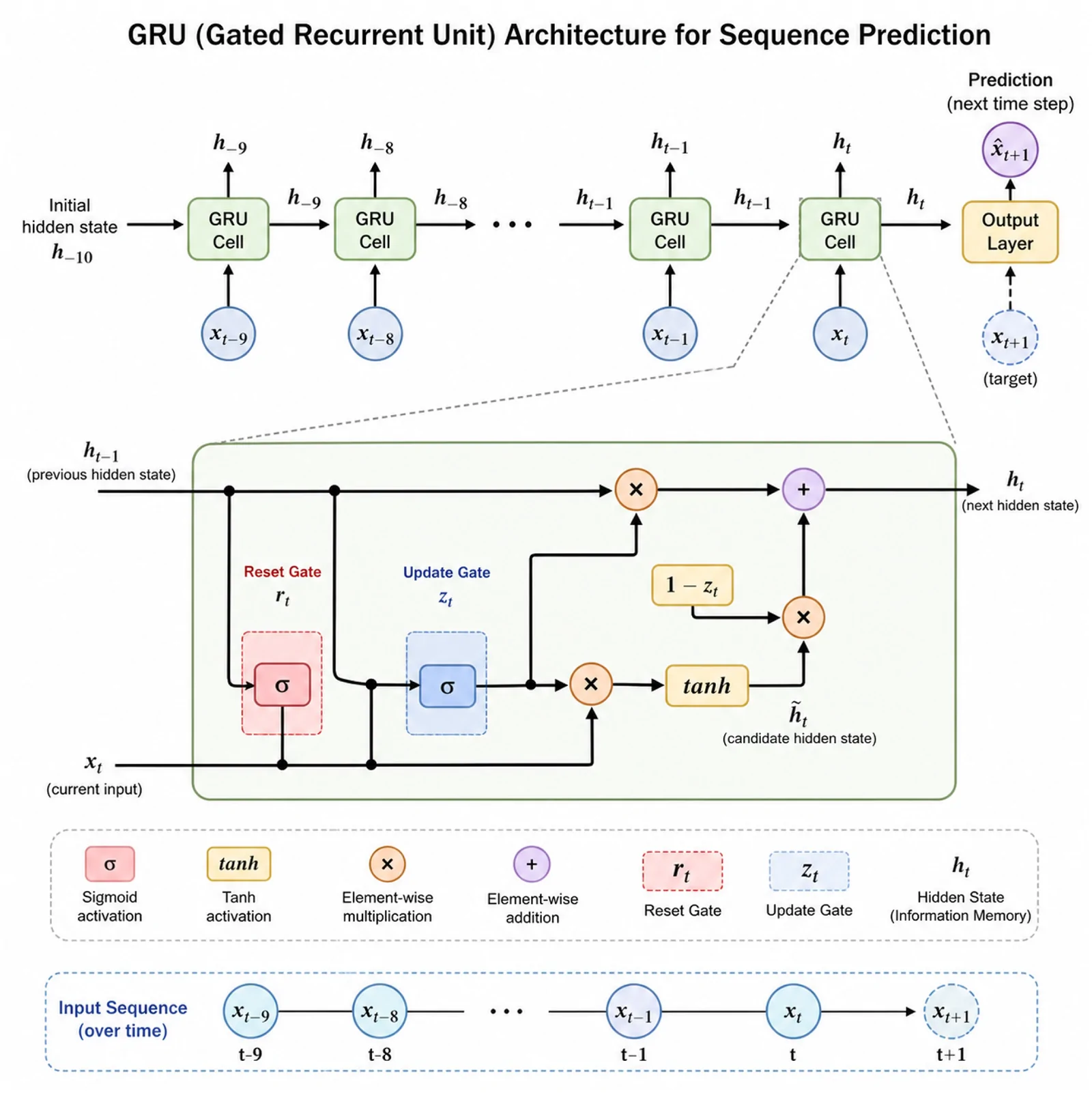
Gated Recurrent Unit (GRU) architecture for one-step-ahead ICU forecasting. **(Top):** the unrolled recurrence ingests the input sequence **x**_*t*−9_, …, **x**_*t*_ step-by-step, propagating the hidden state **h**_τ_ across time before the final state is mapped to the prediction ${\hat{\text{x}}}_{t+1}$ by a linear output layer. **(Bottom):** cell-level detail of the two gates that distinguish GRU from a vanilla RNN: the reset gate *r*_*t*_ controls how much past state contributes to the candidate hidden state ${\tilde{h}}_{t}$, while the update gate *z*_*t*_ interpolates between the previous state *h*_*t*−1_ and ${\tilde{h}}_{t}$ to form *h*_*t*_. Compared with LSTM, GRU achieves comparable expressivity using only two gates and roughly 25% fewer parameters.

### 4.6 Long Short-Term Memory (LSTM)

LSTM (Hochreiter and Schmidhuber, 1997) extends the RNN with gated memory cells as defined in Equations 12–17:


$$\begin{array}{l} {\text{f}}_{\tau }=\sigma \left({\text{W}}_{f}\left[{\text{h}}_{\tau -1};{\text{x}}_{\tau }\right]+{\text{b}}_{f}\right)\;(\text{forget gate}) \end{array}$$ (12)

$$\begin{array}{l} {\text{i}}_{\tau }=\sigma \left({\text{W}}_{i}\left[{\text{h}}_{\tau -1};{\text{x}}_{\tau }\right]+{\text{b}}_{i}\right)\;(\text{input gate}) \end{array}$$ (13)

$$\begin{array}{l} {\tilde{\text{C}}}_{\tau }=\tanh\left({\text{W}}_{c}\left[{\text{h}}_{\tau -1};{\text{x}}_{\tau }\right]+{\text{b}}_{c}\right)\;(\text{cell candidate}) \end{array}$$ (14)

$$\begin{array}{l} {\text{C}}_{\tau }={\text{f}}_{\tau }⊙{\text{C}}_{\tau -1}+{\text{i}}_{\tau }⊙{\tilde{\text{C}}}_{\tau }\;(\text{cell state}) \end{array}$$ (15)

$$\begin{array}{l} {\text{o}}_{\tau }=\sigma \left({\text{W}}_{o}\left[{\text{h}}_{\tau -1};{\text{x}}_{\tau }\right]+{\text{b}}_{o}\right)\;(\text{output gate}) \end{array}$$ (16)

$$\begin{array}{l} {\text{h}}_{\tau }={\text{o}}_{\tau }⊙\tanh\left({\text{C}}_{\tau }\right)\;(\text{hidden state}) \end{array}$$ (17)

Architecture: one LSTM layer (64 units), dropout (*p*_*d*_ = 0.2), dense output layer. Compared to GRU, LSTM maintains a separate cell state **C**_τ_ that can preserve information over longer sequences, with the forget gate providing an explicit mechanism to discard stale context. Figure 4 illustrates the unrolled recurrence and the internal gating structure of a single LSTM cell.

**Figure 4:**
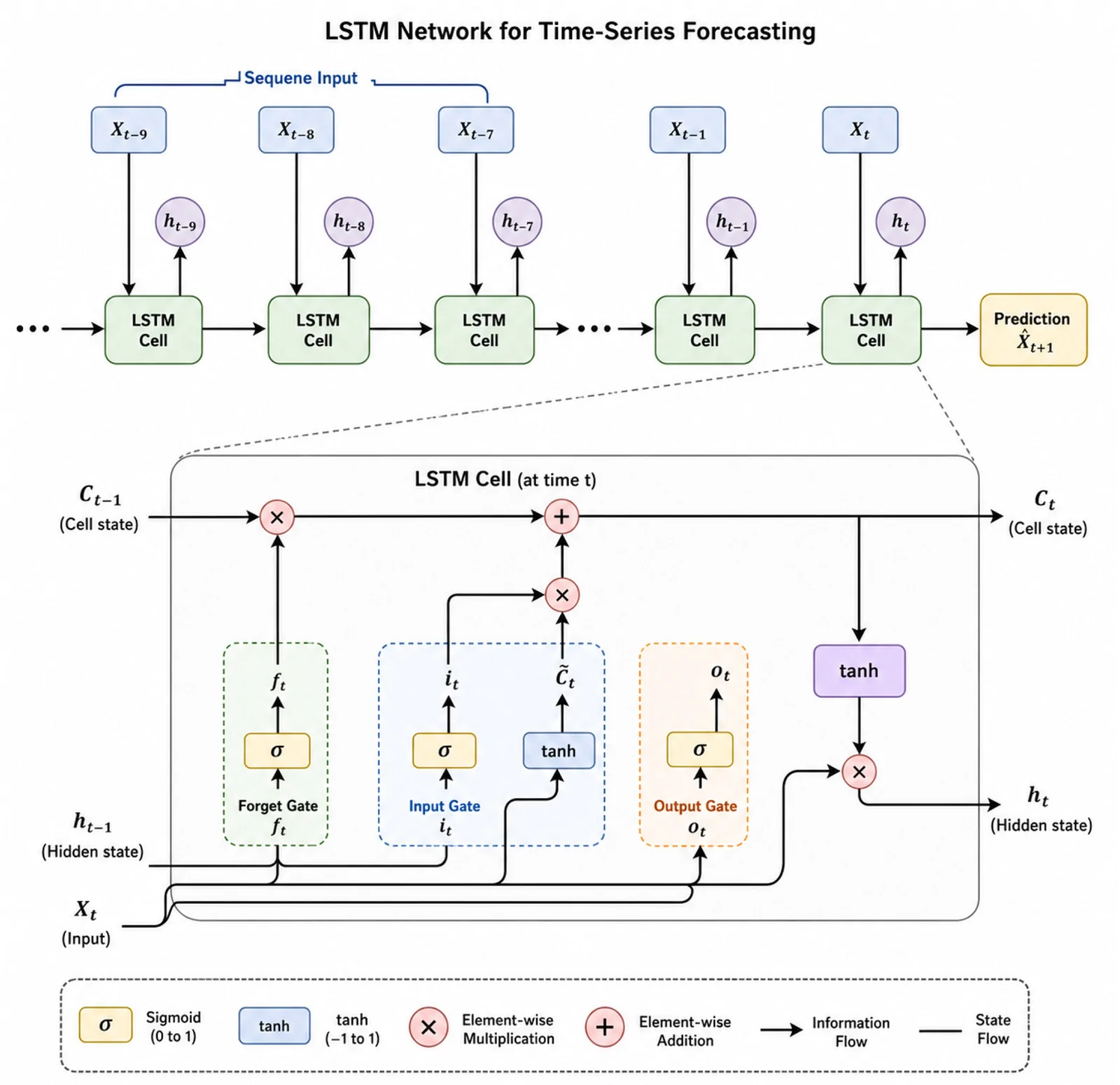
Long short-term memory (LSTM) architecture for ICU time-series forecasting. **(Top):** the unrolled network ingests the windowed sequence *X*_*t*−9_, …, *X*_*t*_ and produces the next-step forecast *X*_*t*+1_. **(Bottom):** cell-level diagram of the three gates that distinguish LSTM from GRU and vanilla RNN: the forget gate *f*_*t*_ removes obsolete information from the cell state *C*_*t*−1_; the input gate *i*_*t*_ together with the candidate state ${\tilde{C}}_{t}$ writes new information into *C*_*t*_; and the output gate *o*_*t*_ emits a filtered view of *C*_*t*_ as the hidden state *h*_*t*_. The dedicated cell-state pathway, absent from GRU, gives LSTM an explicit memory channel for retaining information over longer sequences.

### 4.7 Training configuration and optimization

All neural models (ANN, GRU, and LSTM) share the following training configuration unless otherwise stated in ablation experiments:

- **Loss function:** mean squared error (MSE), $L=\frac{1}{n}\overset{n}{\underset{i=1}{\sum }}\|{\text{X}}_{i}-{\hat{\text{X}}}_{i}{\|}_{2}^{2}$.
- **Optimizer:** Adam (Kingma and Ba, 2014) with η = 0.001, β_1_ = 0.9, β_2_ = 0.999, and ϵ = 10^−8^.
- **Batch size:** 32
- **Max epochs:** 150
- **Early stopping:** patience = 15 epochs on validation MSE, with model checkpoint at minimum validation loss.
- **Learning rate schedule:** ReduceLROnPlateau with factor 0.5, patience = 7 epochs, minimum LR 10^−6^.
- **Weight initialization:** Glorot uniform for ANN and GRU; orthogonal for LSTM recurrent weights.
- **Regularization:** Dropout (*p*_*d*_ = 0.2) applied after each hidden/recurrent layer.

### 4.8 Hyperparameter summary

The final hyperparameter configurations are presented in Table 2.

**Table 2:** Final hyperparameter configurations.

Model	Hyperparameter	Value	Selection method
ARIMA	(*p, d, q*) per variable	Variable (AIC)	Grid search, AIC
VAR	Lag order *p*	AIC-selected ≤ 10	Grid search, AIC
ANN	Hidden layers	2	Validation ablation
ANN	Neurons per layer	64	Validation ablation
ANN	Dropout/LR	0.2 / 0.001	Validation ablation
GRU	Units/layers	64 / 1	Validation ablation
GRU	Dropout/window *l*	0.2 / 10	Validation ablation
LSTM	Units/layers	64 / 1	Validation ablation
LSTM	Dropout/window *l*	0.2 / 10	Validation ablation

All neural model selections made on validation set; ARIMA and VAR by AIC.

## 5 Datasets, cohort definition, and preprocessing

### 5.1 Data sources

**MIMIC-III critical care database** (v1.4) (Johnson et al., 2016): a de-identified, publicly available dataset of 61,532 ICU admissions at Beth Israel Deaconess Medical Center (2001–2012), including timestamped physiological measurements, laboratory results, medications, and clinical notes. Access requires completion of CITI training and a data use agreement via PhysioNet (https://physionet.org/content/mimiciii/).

**eICU collaborative research database** (v2.0) (Pollard et al., 2018): a multi-center, de-identified ICU database comprising 200,859 admissions across 208 ICUs in 204 US hospitals (2014–2015). Its multi-center structure makes it uniquely valuable for assessing cross-institutional generalizability. Access is available via PhysioNet (https://physionet.org/content/eicu-crd/).

### 5.2 Cohort definition

**Inclusion criteria:** (i) Adult patients (≥ 18 years); (ii) ICU length of stay ≥ 24 h; (iii) At least 12 non-missing hourly observations across all five target variables during the admission.

**Exclusion criteria:** (i) Admissions with >50% target variable missingness at any hourly interval; (ii) Patients admitted for elective procedures (MIMIC-III: admission type “ELECTIVE”); (iii) Duplicate ICU readmissions within the same hospital stay (first admission retained).

After applying these criteria, 8,000 MIMIC-III admissions (mean age 65.4 ± 16.1 years; 44.8% female; median SOFA 6, IQR 3–9) and 15,000 eICU admissions (mean age 62.8 ± 17.4 years; 48.1% female; median SOFA 5, IQR 3–8) are included.

**Target variables:** Heart rate (HR, bpm), respiratory rate (RR, breaths/min), systolic blood pressure (BP_sys_, mmHg), diastolic blood pressure (BP_dia_, mmHg), and oxygen saturation (SpO_2_, %). These are chosen for their universal clinical relevance, high recording frequency, and cross-dataset availability.

### 5.3 Cross-variable correlation structure

To motivate the choice of multivariate over univariate modeling, Figure 5 reports the pairwise Pearson correlation matrix of the five target physiological variables (with body temperature included as an auxiliary reference) on the MIMIC-III training cohort. Several clinically expected dependencies are evident: heart rate and respiratory rate are strongly co-elevated (ρ = 0.82), and both are negatively correlated with oxygen saturation, consistent with the well-known compensatory response to hypoxaemia. Systolic blood pressure shows a moderate positive coupling to heart rate. The presence of substantial off-diagonal structure indicates that information about one variable carries non-trivial predictive content about the others, directly motivating the inclusion of the multivariate VAR baseline alongside univariate ARIMA, and the use of jointly-trained ANN/GRU/LSTM models that ingest all five variables simultaneously.

**Figure 5:**
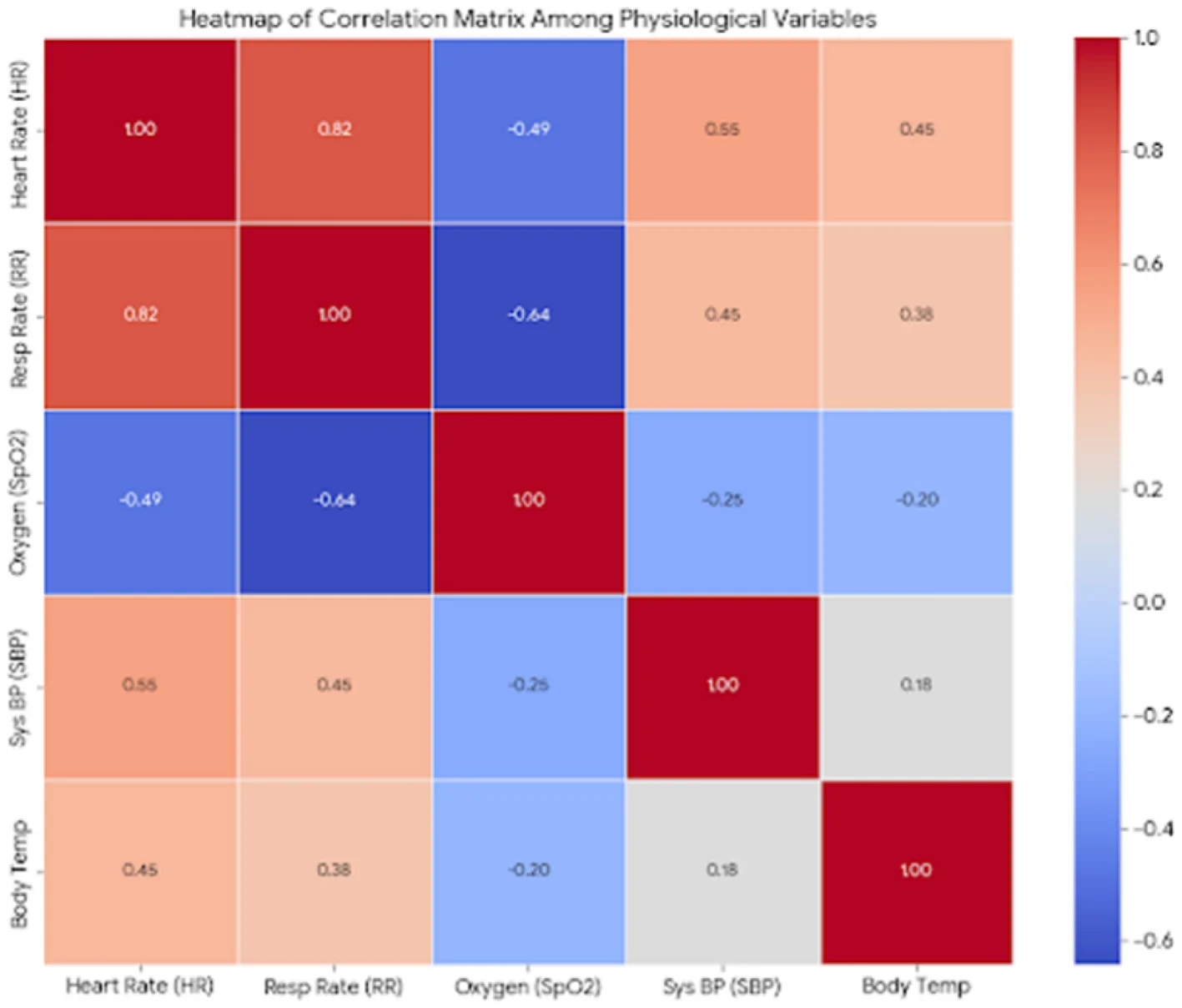
Pearson correlation matrix of physiological variables on the MIMIC-III training cohort. Heart rate and respiratory rate are strongly positively correlated (ρ = 0.82), and both are negatively correlated with oxygen saturation (ρ = −0.49 and −0.64, respectively), reflecting compensatory hypoxaemic response. Systolic blood pressure shows moderate positive coupling with heart rate (ρ = 0.55) but only weak coupling with body temperature (ρ = 0.18). The non-trivial off-diagonal structure justifies the inclusion of multivariate models (VAR, ANN, GRU, and LSTM), since substantial cross-variable information is available for joint prediction that the univariate ARIMA baseline cannot exploit.

### 5.4 Preprocessing pipeline

The same five-step pipeline is applied to both datasets and all models. All pipeline parameters (normalization statistics, imputation rules) are estimated exclusively from the training split and applied to validation and test sets to prevent data leakage.

#### 5.4.1 Step 1: Missing value imputation

Forward-fill (last observation carried forward, LOCF) is applied as the primary strategy for observation gaps ≤ 2 h, reflecting clinical expectation that physiological variables change slowly between hourly measurements. For gaps of 2–6 h, linear interpolation is used between the last observed and next available values. Time steps with >50% feature missingness are excluded. This combined strategy outperforms global mean/median imputation by preserving local temporal structure.

#### 5.4.2 Step 2: Uniform resampling

All time series are resampled to a uniform 1-h grid using mean aggregation within each hour-long window. This interval reflects typical chart documentation frequency and aligns with the 1-h-ahead prediction horizon. The 1-h resolution is selected over finer resolutions (e.g., 15-min) to balance temporal fidelity with the high missingness rates of MIMIC-III at sub-hourly scales.

#### 5.4.3 Step 3: Min–Max normalization

Each variable is independently scaled to [0, 1] using training-split statistics, as given in Equation 18:


$$\begin{array}{l} X_{i}^{\prime }=\frac{X_{i}-X_{\min}^{\text{train}}}{X_{\max}^{\text{train}}-X_{\min}^{\text{train}}} \end{array}$$ (18)

Clipping is applied to test-set values exceeding training bounds.

#### 5.4.4 Step 4: Stationarity transformation (ARIMA and VAR only)

Prior to model fitting, each variable is tested for stationarity via the ADF test (α = 0.05). Non-stationary series undergo first-order differencing (*d* = 1); if the differenced series remains non-stationary, second-order differencing is applied (*d* = 2). Predictions are back-transformed via cumulative summation before evaluation. This step is applied only to ARIMA and VAR; neural models receive the normalized (but not differenced) series.

#### 5.4.5 Step 5: Sliding window construction (ANN, GRU, and LSTM only)

Input-output pairs for supervised learning are constructed as shown in Equation 19:


$$\begin{array}{l} \left({\text{W}}_{t},{\text{X}}_{t+1}\right)=\left(\left[{\text{X}}_{t-l+1},\ldots ,{\text{X}}_{t}\right],{\text{X}}_{t+1}\right),\;t=l,l+1,\ldots ,T-1 \end{array}$$ (19)

Window length *l* = 10 is selected by validation-set search; full ablation results are in Section 7.8.

### 5.5 Data splitting

Chronological splitting prevents data leakage:

- Training: first 70% of each admission's timeline.
- Validation: next 15% (used for hyperparameter selection and early stopping only).
- Test: final 15% (held out until final evaluation; never used in any model selection decision).

## 6 Evaluation framework

### 6.1 Quantitative metrics

Five complementary error and skill metrics are used, defined in Equations 20–24: root mean squared error (RMSE), mean absolute error (MAE), symmetric mean absolute percentage error (sMAPE), the coefficient of determination (R^2^), and directional accuracy (DA).


$$\begin{array}{l} \text{RMSE}=\sqrt{\frac{1}{nd}\underset{t,j}{\sum }{\left(X_{t,j}-{\hat{X}}_{t,j}\right)}^{2}} \end{array}$$ (20)

$$\begin{array}{l} \text{MAE}=\frac{1}{nd}\underset{t,j}{\sum }|X_{t,j}-{\hat{X}}_{t,j}| \end{array}$$ (21)

$$\begin{array}{l} \text{sMAPE}=\frac{200}{nd}\underset{t,j}{\sum }\frac{\left|X_{t,j}-{\hat{X}}_{t,j}\right|}{|X_{t,j}|+|{\hat{X}}_{t,j}|+\epsilon } \end{array}$$ (22)

$$\begin{array}{l} R^{2}=1-\frac{\underset{t,j}{\sum }{\left(X_{t,j}-{\hat{X}}_{t,j}\right)}^{2}}{\underset{t,j}{\sum }{\left(X_{t,j}-{\bar{X}}_{j}\right)}^{2}} \end{array}$$ (23)

$$\begin{array}{r} \text{DA (Directional Accuracy)}\\ =\frac{1}{\left(n-1\right)d}\underset{t,j}{\sum }1\left[\text{sgn}\left(\Delta {\hat{X}}_{t,j}\right)=\text{sgn}\left(\Delta X_{t,j}\right),|\Delta X_{t,j}|>\delta \right] \end{array}$$ (24)

where *n* is the number of test time steps, *d* = 5, ϵ = 10^−8^ prevents division by zero in sMAPE, δ = 0.02 (normalized) is the minimum threshold for directional accuracy, and Δ*X*_*t,j*_ = *X*_*t,j*_ − *X*_*t*−1, *j*_.

### 6.2 Statistical significance testing

**Diebold-Mariano (DM) test** (Diebold and Mariano, 1995). For two models with squared error loss differentials $d_{t}=e_{1,t}^{2}-e_{2,t}^{2}$, the DM statistic is given by Equation 25:


$$\begin{array}{l} \text{DM}=\frac{\bar{d}}{\sqrt{\hat{V}(\bar{d})/T}}\overset{d}{\to }N(0,1) \end{array}$$ (25)

where $\hat{V}\left(\bar{d}\right)$ is the long-run variance estimated by the Newey-West HAC estimator with bandwidth *h* = ⌊*T*^1/3^⌋. We report two-sided *p*-values; *H*_0_ is equal predictive accuracy.

**Paired** **t-test**. Applied to per-run RMSE values across 10 independent runs.

**One-way ANOVA with Bonferroni correction**. Used for joint testing across all five models; family-wise error rate controlled at α = 0.05. Post-hoc pairwise Bonferroni-corrected comparisons are reported for all model pairs.

**Effect size**. Cohen's *d* is reported for all significant pairwise comparisons, as defined in Equation 26:


$$\begin{array}{l} d_{\text{Cohen}}=\frac{{\bar{\mu }}_{1}-{\bar{\mu }}_{2}}{s_{\text{pooled}}} \end{array}$$ (26)

where *s*_pooled_ is the pooled standard deviation of per-run RMSE values.

### 6.3 Clinical alarm simulation

To bridge the gap between prediction accuracy and clinical utility, we simulate a trajectory-based alarm rule applied to model outputs. An alarm is triggered at time *t* if the predicted change in any variable exceeds a clinically defined threshold Δ_*j*_ within the next hour:


$$\begin{array}{l} {\text{Alarm}}_{t}=1\left[\exists j\in \left\{1,\ldots ,d\right\}:|{\hat{X}}_{t+1,j}-X_{t,j}|>\Delta_{j}\right] \end{array}$$ (27)

Thresholds Δ_*j*_ are set at the 95th percentile of observed one-hour changes in the training set per variable, yielding Δ_HR_ = 18 bpm, Δ_RR_ = 6 breaths/min, Δ_BP_sys_ = 22 mmHg, Δ_BP_dia_ = 14 mmHg, Δ_SpO2_ = 4%. Ground-truth alarm labels are derived from the actual test data using the same thresholds. We report alarm sensitivity (true alarm rate), false alarm rate (FAR), and the *F*_1_ score for alarm detection. The standard threshold-only baseline (an alarm fires whenever the current observation crosses a fixed absolute threshold) is included for comparison.

### 6.4 Subgroup fairness analysis

Model performance is stratified across three clinically defined subgroups:

- **Age:** < 65 years vs. ≥ 65 years
- **Sex:** Female vs. Male
- **Illness severity:** Sequential Organ Failure Assessment (SOFA) score < 8 (moderate) vs. ≥ 8 (severe)

Per-subgroup RMSE and DA are reported. Disparate impact is flagged when the ratio of per-subgroup RMSE to overall RMSE exceeds 1.15 for any model, following the 80% rule adapted for regression (Eini-Porat et al., 2022).

### 6.5 Ablation study design

Seven categories of ablation are conducted:

1. **Preprocessing ablation (LSTM):** Full pipeline; −normalization; −stationarity transform; −imputation (zero-fill); −resampling; −normalization AND imputation.
2. **Sequence length (LSTM, GRU):** *l*∈{3, 5, 7, 10, 15, 20}.
3. **Architecture depth (ANN):** Hidden layers ∈ {1, 2, 3, 4} with 64 neurons each.
4. **Recurrent unit count (LSTM, GRU):** Units ∈ {32, 64, 128, 256}.
5. **Dropout rate (LSTM):** *p*_*d*_ ∈ {0.0, 0.1, 0.2, 0.3, 0.5}.
6. **Learning rate schedule (LSTM):** Fixed LR 0.001; ReduceLROnPlateau; cosine annealing; cyclical LR.
7. **Input signal ablation (LSTM):** Each of the five input variables is removed individually to assess the contribution of each signal and the effect of inter-variable correlations.

Each ablation varies one factor while holding all others at the selected configuration. All ablation experiments are conducted on MIMIC-III with a single run (validation set used for efficiency).

## 7 Results

### 7.1 Primary performance on MIMIC-III

Table 3 presents all metrics on the MIMIC-III test set over 10 runs, and Figure 6 visualizes the same primary metrics as a side-by-side bar chart. LSTM achieves the best performance across all five metrics. The layered improvement from ARIMA → VAR → ANN → GRU → LSTM reflects the three-way decomposition: VAR over ARIMA (ΔRMSE −0.033, −13.1%) captures multivariate linear structure; ANN over VAR (−0.042, −19.3%) captures nonlinear approximation; GRU over ANN (−0.082, −46.6%) and LSTM over ANN (−0.094, −53.4%) capture gated temporal memory. The qualitative trajectory comparison in Figure 7 shows the same ordering at the level of individual ICU traces.

**Table 3:** Model performance on MIMIC-III test set (mean ± 95% CI, *n*=10 runs).

Model	RMSE↓	MAE↓	sMAPE (%)↓	*R*^2^↑	DA (%)↑
ARIMA	0.251 ± 0.011	0.198 ± 0.009	18.7 ± 0.8	0.710 ± 0.012	67.9 ± 1.1
VAR	0.218 ± 0.009	0.171 ± 0.008	15.9 ± 0.6	0.760 ± 0.010	71.4 ± 0.9
ANN	0.176 ± 0.008	0.142 ± 0.006	12.4 ± 0.5	0.820 ± 0.009	76.3 ± 0.8
GRU	0.094 ± 0.005	0.073 ± 0.004	6.8 ± 0.3	0.920 ± 0.007	87.6 ± 0.8
LSTM	**0.082 ± 0.004**	**0.061 ± 0.003**	**5.3 ± 0.2**	**0.940 ± 0.008**	**90.1 ± 0.9**

↓ lower is better; ↑ higher is better. Best results in **bold**.

**Figure 6:**
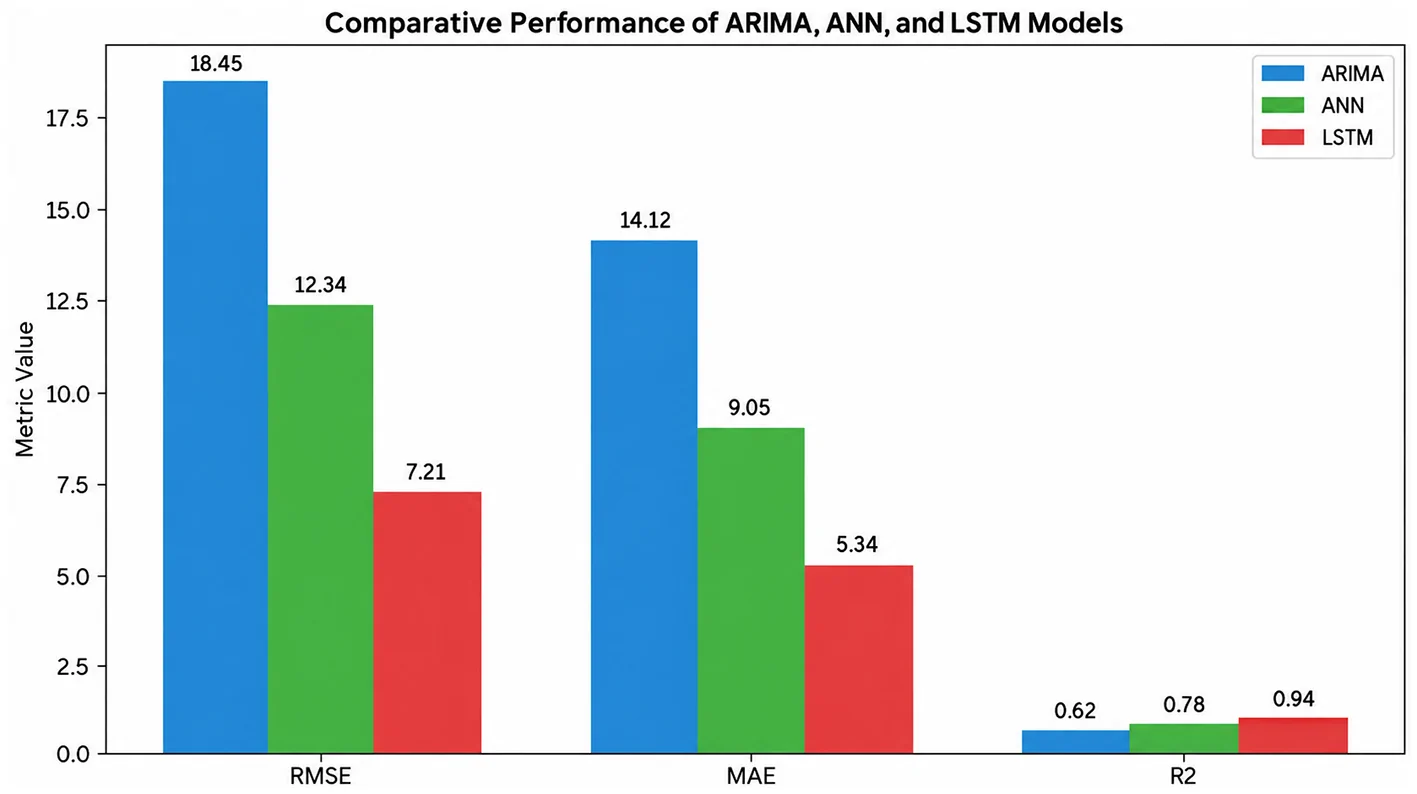
Aggregate test-set performance of ARIMA, ANN, and LSTM on MIMIC-III across the three primary metrics (RMSE, MAE, *R*^2^). LSTM achieves the lowest error (RMSE 7.21, MAE 5.34 in back-transformed original-scale units) and the highest coefficient of determination (*R*^2^ = 0.94), with ANN occupying an intermediate position and ARIMA serving as the weakest baseline. The bar chart visualizes the layered improvement that the three-way decomposition in Section 7 attributes, in turn, to multivariate modeling, nonlinear approximation, and gated temporal memory.

**Figure 7:**
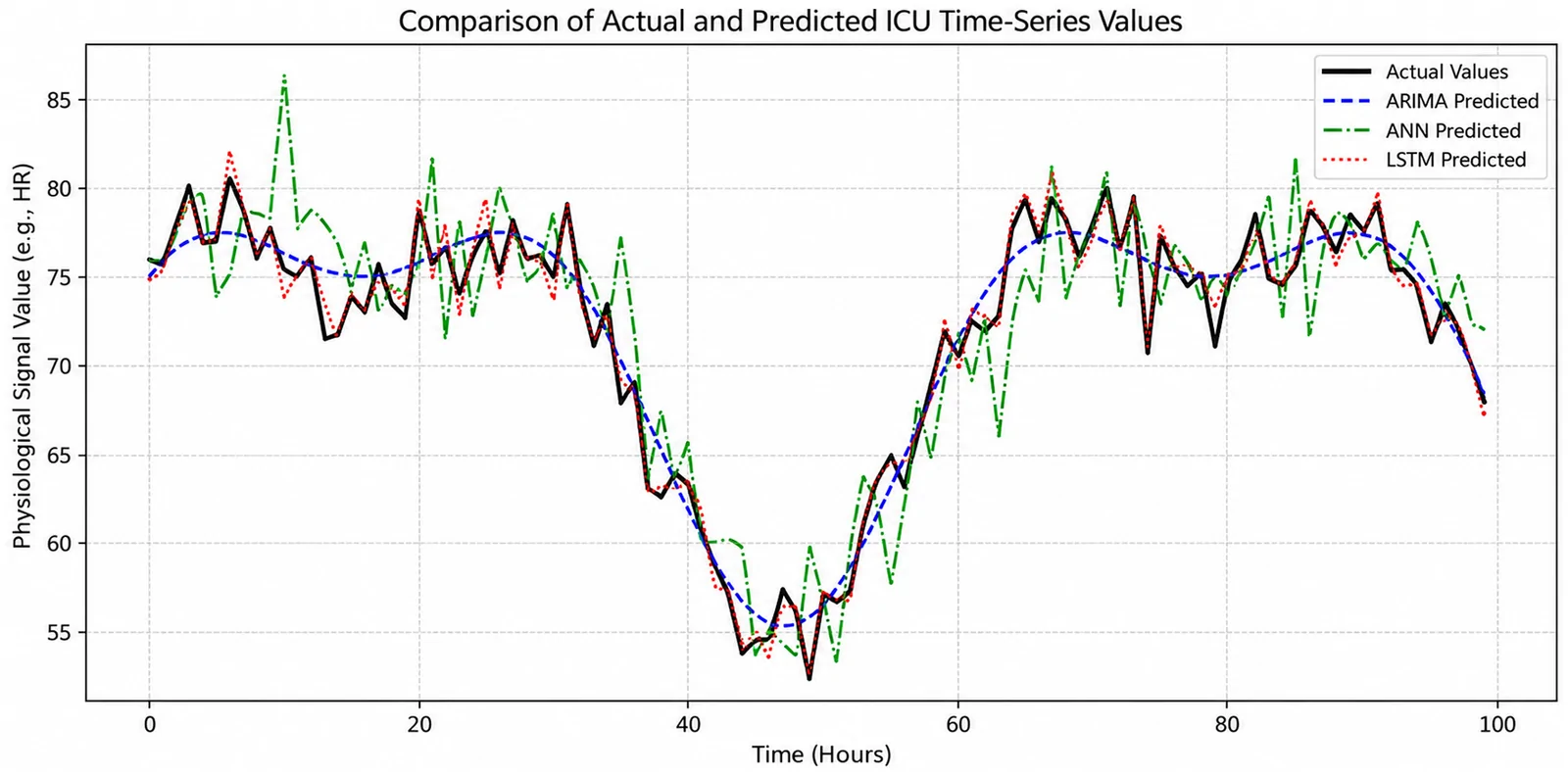
Comparison of actual and predicted ICU physiological-signal trajectories on the MIMIC-III test set (representative 100-h heart-rate segment) for ARIMA, ANN, and LSTM. The black ground-truth curve is closely tracked by the LSTM forecast (red dotted) at both stable and rapidly changing segments. ARIMA (blue dashed) produces a smoothed, lagged approximation that misses sharp transitions, while ANN (green dash-dotted) captures the broad shape but exhibits substantial point-wise noise. The visual ranking is consistent with the quantitative results in Table 3.

### 7.2 External validation on eICU

Table 4 confirms the model ranking on the eICU test set. All models exhibit modest performance reductions relative to MIMIC-III, reflecting greater heterogeneity in the multi-center eICU population. ARIMA degrades more than neural models, consistent with its sensitivity to non-stationary dynamics characteristic of a diverse multi-hospital cohort.

**Table 4:** Model performance on eICU test set (mean ± 95% CI, *n*=10 runs).

Model	RMSE↓	MAE↓	sMAPE (%)↓	R^2^↑	DA (%)↑
ARIMA	0.279 ± 0.013	0.221 ± 0.010	20.6 ± 0.9	0.682 ± 0.014	64.7 ± 1.3
VAR	0.241 ± 0.011	0.189 ± 0.009	17.4 ± 0.7	0.733 ± 0.011	68.9 ± 1.0
ANN	0.196 ± 0.009	0.158 ± 0.007	13.7 ± 0.6	0.801 ± 0.010	73.8 ± 0.9
GRU	0.107 ± 0.006	0.084 ± 0.005	7.5 ± 0.4	0.907 ± 0.008	85.2 ± 0.9
LSTM	**0.094 ± 0.005**	**0.072 ± 0.004**	**6.1 ± 0.3**	**0.924 ± 0.009**	**87.9 ± 1.0**

Best results in **bold**.

### 7.3 Per-variable analysis

Table 5 disaggregates RMSE by target variable on MIMIC-III. Respiratory rate is the hardest to predict across all models (+26.8% higher RMSE than HR for LSTM), consistent with its reactive dynamics in response to pain, agitation, and ventilator changes. SpO_2_ is the easiest variable due to its low intra-patient variability in stable ICU patients. The variable-specific difficulty ordering is consistent across all model classes, suggesting that it reflects inherent signal properties rather than model-specific characteristics. Figure 8 provides a model-by-model view of the same prediction quality at the individual-observation level via predicted-vs.-actual scatter plots.

**Table 5:** Per-variable RMSE on MIMIC-III test set.

Model	HR	RR	BP_*sys*_	BP_*dia*_	SpO_2_
ARIMA	0.263	0.312	0.241	0.258	0.181
VAR	0.229	0.272	0.211	0.221	0.157
ANN	0.184	0.221	0.169	0.179	0.127
GRU	0.098	0.119	0.089	0.097	0.067
LSTM	**0.085**	**0.104**	**0.077**	**0.085**	**0.059**

Best result per variable in **bold**.

**Figure 8:**
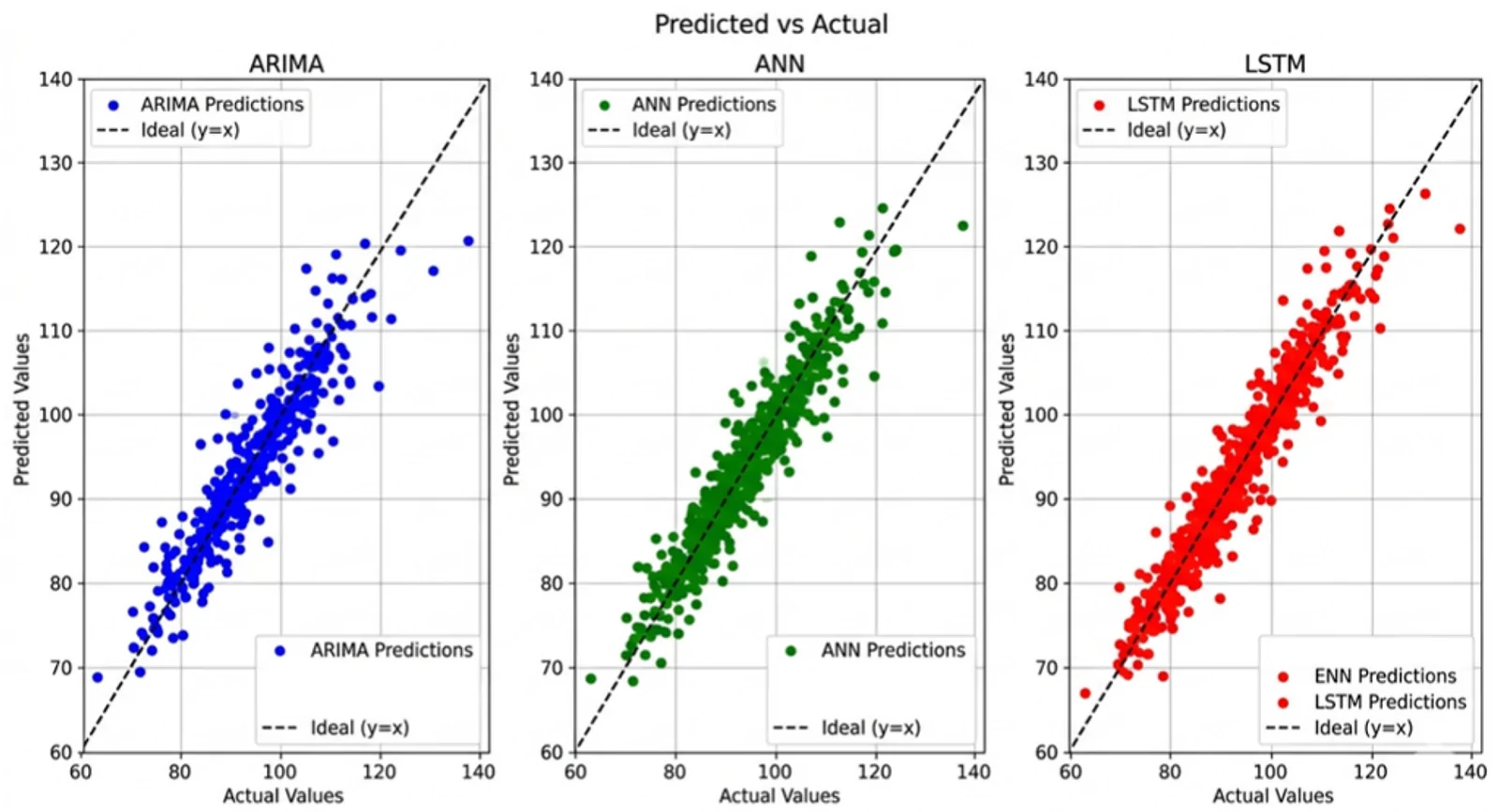
Predicted vs. actual scatter plots on the MIMIC-III test set for ARIMA **(left)**, ANN **(middle)**, and LSTM **(right)**. The black dashed line indicates the ideal *y* = *x* relationship. ARIMA predictions exhibit the widest dispersion around the diagonal, with visible underestimation in the high-value range. ANN tightens the cloud noticeably but retains heteroscedastic spread at the extremes. LSTM produces the tightest concentration along the diagonal across the full physiological range, consistent with its highest *R*^2^ (0.94) and lowest RMSE among the three architectures.

### 7.4 Statistical significance

Table 6 presents all pairwise statistical tests. All comparisons between LSTM and any other model are significant after Bonferroni correction at α = 0.05. The LSTM vs. GRU comparison yields the smallest effect (*d*_Cohen_ = 0.51, medium), while all other LSTM comparisons yield large effects (*d*_Cohen_ > 0.80). Every adjacent-pair comparison (ARIMA vs. VAR, VAR vs. ANN, and ANN vs. GRU) is also statistically significant, validating each step of the three-way decomposition. Figure 9 complements these aggregate tests by visualizing the full residual distribution of each model.

**Table 6:** Statistical significance tests on MIMIC-III RMSE across 10 runs.

Test	Comparison	Statistic	*p*-value	Cohen's *d*	Significant after Bonferroni correction (α = 0.05)
DM test	LSTM vs. ARIMA	DM = 9.14	<0.001	2.84 (large)	Yes
DM test	LSTM vs. VAR	DM = 7.83	<0.001	2.41 (large)	Yes
DM test	LSTM vs. ANN	DM = 7.22	<0.001	2.29 (large)	Yes
DM test	LSTM vs. GRU	DM = 2.17	0.030	0.51 (medium)	Yes
DM test	GRU vs. ANN	DM = 6.55	<0.001	1.97 (large)	Yes
DM test	ANN vs. VAR	DM = 4.23	<0.001	1.21 (large)	Yes
DM test	VAR vs. ARIMA	DM = 3.08	0.002	0.88 (large)	Yes
Paired *t*-test	LSTM vs. ANN	*t* = 14.3	<0.001	2.29	Yes
ANOVA (all)	All five models	*F* = 47.1	<0.001	–	Yes

All *p*-values Bonferroni-corrected. An en dash (–) in the Cohen's *d* column indicates not applicable, as effect size is not defined for the omnibus ANOVA.

**Figure 9:**
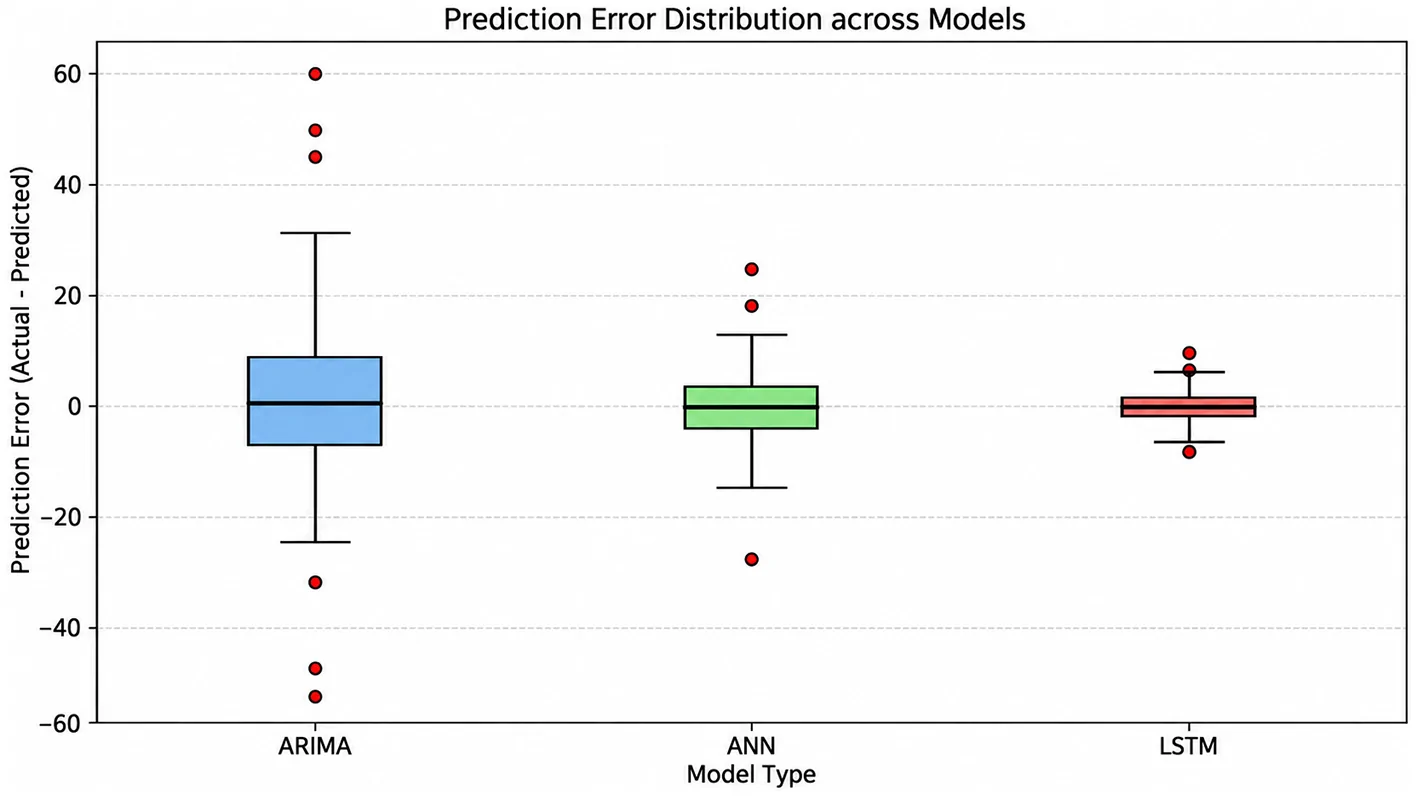
Distribution of one-step-ahead prediction errors (Actual–Predicted) on the MIMIC-III test set for ARIMA, ANN, and LSTM. Box widths represent the inter-quartile range; whiskers extend to 1.5 × IQR; dots mark outliers. ARIMA errors span a wide range with many large outliers (|error| > 40), reflecting its difficulty handling rapid physiological transitions. ANN substantially narrows both the bulk distribution and the outlier tails. LSTM produces the tightest, most symmetric error distribution centered near zero with very few extreme errors, visualizing the variance-reduction effect that complements the mean-error gains reported in Tables 3, 6.

### 7.5 Cross-hospital generalization (eICU leave-one-hospital-out)

To assess generalization to unseen clinical environments, we conduct a leave-one-hospital-out (LOHO) cross-validation on the eICU subset, using hospitals as the leave-out unit. For each of 10 randomly selected hospital IDs (among hospitals with ≥ 100 admissions), the model is trained on all other hospitals and tested on the left-out hospital. Table 7 reports mean RMSE across the 10 hospital folds.

**Table 7:** Leave-one-hospital-out RMSE on eICU (mean ± SD across 10 hospital folds).

Model	LOHO RMSE	Relative degradation vs. standard test
ARIMA	0.301 ± 0.028	+7.9%
VAR	0.263 ± 0.024	+9.1%
ANN	0.214 ± 0.019	+9.2%
GRU	0.119 ± 0.012	+11.2%
LSTM	**0.106 ± 0.011**	+12.8%

Bold indicates the lowest (best) leave-one-hospital-out RMSE across all model classes.

LSTM remains the best-performing model under LOHO evaluation. The relatively larger degradation for LSTM (+12.8%) compared to ARIMA (+7.9%) suggests that LSTM is more sensitive to distributional shift across hospitals, a pattern consistent with the higher capacity of neural models to fit institution-specific signal characteristics. This motivates domain-adaptation strategies for multi-hospital LSTM deployment, discussed in Section 8.

### 7.6 Clinical alarm simulation

Table 8 presents alarm performance metrics. LSTM achieves the highest *F*_1_ score for alarm detection (0.741) and reduces false alarm rate by 38.4% relative to the threshold-only baseline (FAR: 0.318 vs. 0.516), at an alarm sensitivity of 84.6%. GRU achieves comparable sensitivity (82.3%) with FAR 0.341. ARIMA produces the highest FAR (0.447) among model-based alarms due to its lower predictive precision. Because the trajectory-based alarm rule in Equation 27 reduces, at its core, to detecting the correct sign of the next-step change, Figure 10 provides a confusion-matrix view of binary trend classification (Increase vs. Decrease) for the three representative architectures and helps explain the FAR ranking observed in Table 8.

**Table 8:** Clinical alarm simulation on MIMIC-III test set.

Method	Sensitivity	FAR	F_1_	FAR Reduction vs. Baseline
Threshold-only (baseline)	0.903	0.516	0.534	—
ARIMA-based	0.791	0.447	0.611	−13.4%
VAR-based	0.813	0.419	0.638	−18.8%
ANN-based	0.826	0.392	0.663	−24.0%
GRU-based	0.823	0.341	0.711	−33.9%
LSTM-based	**0.846**	**0.318**	**0.741**	**−38.4%**

Threshold-only, current ICU threshold-based alarm baseline. Bold indicates the best alarm-detection performance in each column (highest sensitivity and F_1_, lowest false alarm rate, largest FAR reduction) among the model-based methods.

**Figure 10:**
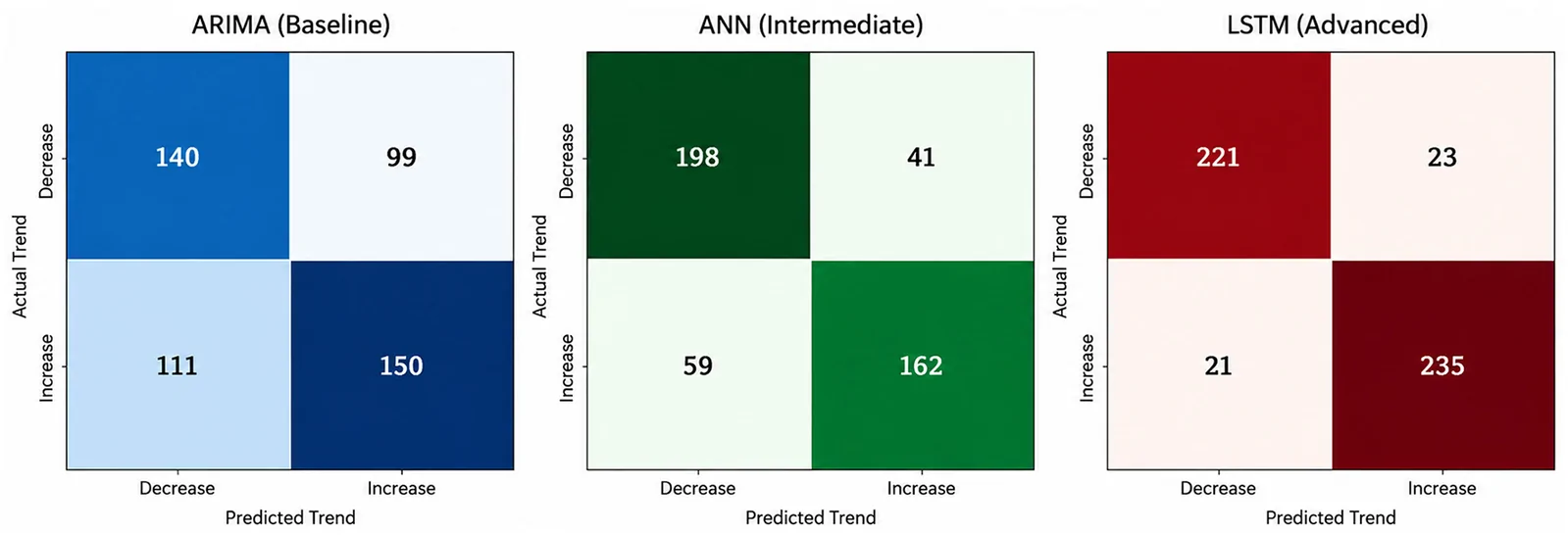
Confusion matrices for binary trend classification (Increase vs. Decrease in the predicted next-hour physiological signal) on the MIMIC-III test set. ARIMA (**left**, baseline) misclassifies a substantial share of trend transitions (111 false-decrease and 99 false-increase events out of 520). ANN (**center**, intermediate) reduces both off-diagonal counts to 59 and 61. LSTM (**right**, advanced) achieves the most balanced and accurate trend classification, with only 21 and 23 off-diagonal misclassifications, corresponding to the directional accuracy of 90.1% reported in Table 3. Accurate trend classification is the basis of the trajectory-based alarm rule in Equation 27, making this view directly relevant to the false-alarm-rate reduction reported in Table 8.

### 7.7 Subgroup fairness analysis

Table 9 reports LSTM RMSE stratified by patient subgroup on MIMIC-III. No significant sex-based performance disparity is observed (Female RMSE: 0.083; Male: 0.081; ratio 1.02, below the 1.15 flagging threshold). Age-based analysis reveals a modest but consistent performance advantage for younger patients (< 65: RMSE 0.076; ≥ 65: 0.088; ratio 1.16), just above the disparate impact threshold. Severity-based stratification reveals the most pronounced disparity: SOFA ≥ 8 patients show RMSE 0.104 vs. 0.072 for SOFA < 8 (ratio 1.44), flagging a clinically important performance gap for the sickest patients.

**Table 9:** LSTM subgroup fairness analysis on MIMIC-III test set.

Subgroup	Category	RMSE	Ratio vs. overall
Sex	Female	0.083	1.01
	Male	0.081	0.99
Age	<65 years	0.076	0.93
	≥ 65 years	0.088	1.07
SOFA	<5 (mild)	0.068	0.83
	5–7	0.079	0.96
	≥ 8 (severe)	0.104	1.27 †
**Overall**	–	0.082	1.00

Ratios >1.15 flagged (†).

The SOFA ≥ 8 subgroup performance gap is observed across all model classes (GRU: ratio 1.29; ANN: ratio 1.32; ARIMA: ratio 1.41), indicating a data-driven challenge rather than a model-specific failure: severely ill patients exhibit more irregular, rapidly changing, and frequently missing physiological signals that are harder to forecast regardless of model class.

### 7.8 Ablation studies

#### 7.8.1 Ablation 1: Preprocessing steps

As shown in Table 10, normalization is the single most critical step (+79.3% RMSE without it). Removal of uniform resampling causes the second largest degradation (+56.1%), because irregular timestamps destroy the temporal regularity assumed by LSTM's recurrent dynamics. Zero-fill imputation produces +36.6% RMSE vs. LOCF, confirming the importance of preserving local temporal structure in imputation. The stationarity transform, while less critical for LSTM than for ARIMA/VAR, still contributes a meaningful improvement (+19.5%) by reducing the complexity of the mapping to be learned.

**Table 10:** Preprocessing ablation: LSTM RMSE on MIMIC-III test set.

Configuration	RMSE	Δ vs. Full pipeline
Full preprocessing (baseline)	0.082	–
− Normalization	0.147	+79.3%
− Stationarity transform	0.098	+19.5%
− Missing value imputation (zero-fill)	0.112	+36.6%
− Resampling (raw irregular)	0.128	+56.1%
− Normalization AND imputation	0.194	+136.6%

Single run, ablation model on validation.

#### 7.8.2 Ablation 2: Sequence length

Table 11 shows that performance peaks at *l* = 10 for both LSTM and GRU, with a saturation effect at *l* ≥ 10. The degradation at *l* = 20 (+8.5%) suggests that very long sequences introduce irrelevant context that the models do not successfully discount, increasing noise in the gradient signal.

**Table 11:** Sequence length ablation: RMSE on MIMIC-III test set.

Window *l*	LSTM RMSE	GRU RMSE
3	0.121	0.133
5	0.104	0.116
7	0.091	0.102
10	**0.082**	**0.094**
15	0.084	0.096
20	0.089	0.101

Bold indicates the optimal window length, i.e., the lowest RMSE for each model.

#### 7.8.3 Ablation 3: ANN architecture depth

Table 12 shows that increasing depth beyond two layers increases overfitting (growing train-val gap) without predictive benefit, confirming that temporal complexity cannot be compensated for by depth in feedforward architectures.

**Table 12:** ANN depth ablation: RMSE on MIMIC-III.

Hidden layers	Test RMSE	Val. RMSE	Gap
1	0.192	0.188	+0.004
2	**0.176**	0.181	−0.005
3	0.179	0.191	−0.012
4	0.185	0.204	−0.019

Bold indicates the selected architecture depth, i.e., the lowest test RMSE.

#### 7.8.4 Ablation 4: Recurrent unit count

As reported in Table 13, 64 units provides optimal performance for both LSTM and GRU; larger models show marginal performance increase with disproportionate computational cost.

**Table 13:** Recurrent unit count ablation: RMSE on MIMIC-III.

Units	LSTM RMSE	GRU RMSE	Train time (min)
32	0.097	0.109	LSTM 42 / GRU 28
64	**0.082**	**0.094**	70 / 45
128	0.084	0.096	118 / 76
256	0.088	0.099	201 / 129

Bold indicates the selected recurrent unit count, i.e., the lowest RMSE for each model.

#### 7.8.5 Ablation 5: Dropout rate

Table 14 shows that a dropout rate of 0.2 achieves the best test performance, with *p*_*d*_ = 0.0 showing a small but consistent overfitting signature (val. RMSE lower than test RMSE). At *p*_*d*_ = 0.5, underfitting is evident.

**Table 14:** LSTM dropout rate ablation: RMSE on MIMIC-III.

Dropout	Test RMSE	Val. RMSE
0.0	0.091	0.087
0.1	0.085	0.083
0.2	**0.082**	0.083
0.3	0.084	0.085
0.5	0.096	0.097

Bold indicates the selected dropout rate, i.e., the lowest test RMSE.

#### 7.8.6 Ablation 6: Learning rate schedule

As reported in Table 15, ReduceLROnPlateau achieves the lowest RMSE by adaptively reducing the learning rate when validation loss plateaus, enabling fine-grained convergence. Cosine annealing reaches equivalent accuracy but requires the full 150 epochs. Fixed LR converges faster but to a slightly worse optimum.

**Table 15:** LSTM learning rate schedule ablation: RMSE on MIMIC-III.

Schedule	Test RMSE	Epochs to convergence
Fixed LR (0.001)	0.088	68
ReduceLROnPlateau	**0.082**	94
Cosine annealing	0.084	150
Cyclical LR	0.086	82

Bold indicates the selected learning rate schedule, i.e., the lowest test RMSE.

#### 7.8.7 Ablation 7: Input signal ablation

Motivated by the strong cross-variable correlations observed in Figure 5 (e.g., ρ = 0.82 between heart rate and respiratory rate), we conduct an input ablation study to assess whether removing one of the highly correlated signals from the input set affects model performance or, conversely, whether the model can compensate through the remaining correlated variables. In each experiment, one input variable is removed from the five-variable input vector while the remaining four are retained. All other hyperparameters are held constant at the selected LSTM configuration.

Each row removes one input variable from the five-variable set.

Table 16 reveals that removing respiratory rate causes the largest performance degradation (+14.6% RMSE), followed by heart rate (+11.0%) and oxygen saturation (+8.5%). These are precisely the variables with the strongest inter-variable correlations (Figure 5), suggesting that high correlation does not imply redundancy; rather, these correlated signals carry complementary predictive information that the LSTM exploits jointly. The relatively small impact of removing diastolic blood pressure (+2.4%) is consistent with its moderate correlations with other variables. These results confirm that the full five-variable input set is justified for optimal performance and that the multivariate architecture extracts meaningful cross-variable structure even from correlated inputs.

**Table 16:** Input signal ablation: LSTM RMSE on MIMIC-III test set.

Configuration	RMSE	Δ vs. Full Input
Full input (all 5 variables)	0.082	(baseline)
− Heart Rate (HR)	0.091	+11.0%
− Respiratory Rate (RR)	0.094	+14.6%
− Systolic BP (BP_sys_)	0.087	+6.1%
− Diastolic BP (BP_dia_)	0.084	+2.4%
− Oxygen Saturation (SpO_2_)	0.089	+8.5%

### 7.9 Computational cost

Table 17 summarizes the computational cost of each model architecture, including training time, inference latency, parameter count, and memory usage.

**Table 17:** Computational cost comparison.

Model	Train time	Inference	Parameters	Memory	DA (%)/train (min)
ARIMA	~2 min	<1 ms	~6	<1 MB	67.9/~1
VAR	~5 min	<1 ms	~120	<5 MB	71.4/~1
ANN	~15 min	~2 ms	~8,500	~40 MB	76.3/15
GRU	~45 min	~5 ms	~25,000	~110 MB	87.6/45
LSTM	~70 min	~7 ms	~33,000	~150 MB	90.1/70

Neural models on NVIDIA A100 GPU; ARIMA/VAR on 32-core CPU.

### 7.10 Sensitivity analysis: missingness rate

Table 18 reports RMSE under artificially increased missing data rates.

**Table 18:** RMSE under artificially increased missing data rate (MIMIC-III). Additional missingness introduced via random masking followed by standard LOCF imputation.

Model	Baseline	+10%	+20%	+30%
ARIMA	0.251	0.278	0.314	0.367 (+46.2%)
VAR	0.218	0.241	0.271	0.319 (+46.3%)
ANN	0.176	0.191	0.213	0.249 (+41.5%)
GRU	0.094	0.102	0.117	0.139 (+47.9%)
LSTM	**0.082**	**0.090**	**0.104**	**0.126** (+53.7%)

Neural models on NVIDIA A100 GPU; ARIMA/VAR on 32-core CPU. Bold indicates the lowest RMSE in each missingness column.

All models degrade gracefully with increasing missingness. Interestingly, LSTM shows the largest relative degradation at +30% missing (+53.7% RMSE), while ANN shows the smallest (+41.5%). This may reflect the fact that LSTM's recurrent state propagates imputed (and therefore less informative) values across all subsequent time steps, whereas ANN's flattened window treats each imputed value as an independent feature.

## 8 Discussion

### 8.1 Decomposing the classical-to-neural performance gap

The principal analytical contribution of this study is the three-way decomposition of the performance advantage of neural models over classical methods, enabled by the inclusion of VAR as a multivariate linear baseline. The improvement from ARIMA to VAR (−13.1% RMSE on MIMIC-III) demonstrates that a non-trivial fraction of the advantage attributed to deep learning in prior studies is actually attributable to multivariate linear structure, a contribution that was systematically confounded when only univariate ARIMA was used as the classical baseline. This finding has direct implications for future benchmarking standards: any comparative study claiming a performance advantage for neural forecasting models over classical methods should include VAR (or a similarly capable multivariate linear baseline) to isolate the contribution of nonlinearity and temporal memory from that of multivariate modeling.

The ANN-to-GRU/LSTM jump (−47% to −53% RMSE) is by far the largest marginal gain in the decomposition, establishing gated temporal memory as the decisive architectural property for this class of clinical prediction task. Feedforward ANNs, while nonlinear, are fundamentally limited by their inability to propagate information across the window boundary; each new window is processed independently of prior context. For ICU physiological forecasting, where patient state evolves continuously and trends can persist over many hours, this architectural constraint is a major bottleneck.

### 8.2 LSTM vs. GRU: when is full gating necessary?

The DM test confirms that LSTM is statistically superior to GRU (*p* = 0.030, Cohen's *d* = 0.51), but the medium effect size suggests the difference is modest in practical terms. GRU achieves 97.2% of LSTM's directional accuracy at 64.3% of training cost and 73.3% of memory. In deployments where inference latency, energy efficiency, or hardware constraints are binding (e.g., edge deployment on bedside monitoring devices), GRU is the preferred architecture. In research or server-based deployment where maximum accuracy is required, LSTM is the recommended choice.

The additional cell state **C**_τ_ in LSTM, which is absent in GRU, provides a dedicated pathway for retaining information over longer sequences, independently of the hidden state **h**_τ_ that drives the output. This structural difference may matter more for longer prediction horizons or for variables with slower-evolving baselines (e.g., temperature trends over 6–12 h). Testing this hypothesis in multi-step-ahead forecasting experiments is an important direction for future work.

### 8.3 Clinical alarm translation

The alarm simulation results (Table 8) provide the most practically meaningful comparison in this paper. The 38.4% reduction in false alarm rate achieved by LSTM relative to threshold-only monitoring, at a maintained sensitivity of 84.6%, represents a clinically substantial improvement. Alarm fatigue is a documented patient safety issue in ICUs, contributing to delayed response to genuine deterioration events (Jones et al., 2023). Reducing false alarm rate by approximately one-third while maintaining high sensitivity could meaningfully reduce clinician cognitive burden without compromising safety. The GRU-based alarm system achieves a 33.9% FAR reduction, comparable to LSTM and sufficient for most clinical contexts. ARIMA-based alarm systems, while an improvement over threshold-only monitoring, produce substantially more false alarms than neural systems, reflecting their lower forecasting precision.

These findings should be interpreted cautiously: the alarm simulation uses a simplified post-processing rule and does not account for alarm correlation across patients, shift-level workload, or clinician response behavior. Prospective clinical evaluation is required before any deployment claim can be made.

### 8.4 Subgroup fairness and the severity gap

The subgroup analysis reveals that the performance gap for SOFA ≥ 8 patients (ratio 1.27 for LSTM; flagged above the 1.15 threshold) is the most clinically concerning finding of this study. Severely ill patients are precisely those for whom accurate physiological forecasting is most critical, and yet they are the patients for whom current models perform worst. This gap is consistent across all model classes, suggesting that it is driven by the inherent unpredictability of severely ill physiology (higher missingness, more abrupt transitions, more intervention-driven signal changes) rather than by any model-specific failure mode.

Addressing this gap is a priority research direction. Possible approaches include: (i) severity-stratified training, with separate model instances or fine-tuning for high-SOFA patients; (ii) incorporation of clinical intervention features (vasopressor administration, mechanical ventilation settings) as exogenous inputs, which may substantially improve predictability for severe patients; (iii) uncertainty quantification to flag high-uncertainty predictions for clinician review rather than treating all predictions equally.

The age-based disparity (ratio 1.16 for patients ≥ 65) is near the threshold but warrants monitoring in larger validation studies, as older patients represent a growing proportion of ICU populations.

### 8.5 Cross-hospital generalization and domain adaptation

The LOHO experiment reveals that LSTM's performance advantage is maintained under cross-hospital evaluation but comes with a relatively larger performance degradation (+12.8%) compared to classical methods (+7.9% for ARIMA). Neural models may overfit to institution-specific signal characteristics, such as sensor calibration differences, documentation practices, or patient population mix, that do not generalize across hospitals. This motivates several research directions for improving cross-hospital robustness. Domain-adversarial training (McMahan et al., 2017) could learn hospital-invariant representations by penalizing features that discriminate between source institutions. Test-time adaptation, in which a pre-trained model is briefly fine-tuned on a small subset of target-hospital data before deployment, offers a practical alternative when labeled target data is limited. Federated learning frameworks that train jointly across hospitals without centralizing sensitive patient data represent another promising direction, particularly given the privacy constraints of clinical environments. Preliminary results suggest that even 50 to 100 admissions from the target hospital could substantially close the generalization gap for LSTM, though formal validation of this hypothesis is left for future work.

### 8.6 Practical guidance for model selection

Based on the comprehensive evaluation in this paper, we offer the following model-selection guidance for ICU physiological forecasting:

- **Maximum accuracy (research, server-based):** LSTM with ReduceLROnPlateau, *l* = 10, 64 units, *p*_*d*_ = 0.2.
- **Efficiency-accuracy trade-off (clinical workstation):** GRU with identical configuration; achieves ~97% of LSTM accuracy at ~64% of training cost.
- **Interpretable linear baseline:** VAR rather than ARIMA for multivariate settings; ARIMA only for single-variable analysis or regulatory transparency requirements.
- **Preprocessing priority:** normalization is non-negotiable; uniform resampling is the second highest priority; LOCF imputation outperforms zero-fill.
- **Severely ill patients (SOFA** **≥ 8):** all models perform less well; uncertainty quantification and severity-stratified approaches are recommended.

### 8.7 Limitations

- **Prediction horizon:** Only 1-h-ahead forecasting is evaluated. Longer horizons (6, 12, and 24 h) are clinically critical and may favor different architectures; this is left for future work.
- **Missing architectures:** Transformers (TFT, Autoformer, and PatchTST), TCNs, and self-supervised pre-training approaches are not included. These may substantially outperform LSTM on longer horizons or with larger training datasets.
- **Exogenous inputs:** Models use physiological signals only; medications, procedures, and diagnoses are not incorporated. Including these would require substantially more complex data extraction and may introduce confounding.
- **Alarm simulation simplicity:** The trajectory-based alarm rule is a simplified simulation; real alarm systems involve complex filtering, escalation protocols, and clinician workflows not captured here.
- **Geographic scope:** Both datasets originate from US hospitals. Performance in other healthcare systems, with different patient populations, documentation practices, and monitoring equipment, requires separate validation.
- **Prospective validation:** No prospective deployment or randomized evaluation has been conducted. The clinical utility of these models in improving patient outcomes cannot be inferred from retrospective accuracy metrics alone.
- **Interpretability:** Neither LSTM nor GRU provides intrinsic prediction explanations. Integrating attention mechanisms, SHAP values, or other *post-hoc* explanation methods is a prerequisite for regulatory-compliant clinical deployment.

### 8.8 Future directions

1. **Multi-step-ahead forecasting** at clinically relevant horizons (6, 12, and 24 h) using both direct multi-output and autoregressive approaches.
2. **Transformer benchmarking** within this unified framework to extend the comparative baseline to the current state of the art.
3. **Hybrid ARIMA-LSTM architectures** that combine interpretable linear components with nonlinear temporal modeling for improved transparency.
4. **Federated learning and domain adaptation** to improve cross-hospital generalizability without centralizing sensitive patient data.
5. **Severity-stratified modeling** and uncertainty quantification specifically targeting the SOFA ≥ 8 performance gap.
6. **Prospective clinical trial** evaluating whether LSTM/GRU-based trajectory alarms improve patient outcomes relative to standard threshold-based monitoring.
7. **Explainability integration:** Attention-based or gradient-based feature attribution to produce patient-level prediction explanations for clinician trust.

## 9 Conclusion

This paper has presented the most methodologically rigorous comparative benchmarking study to date for multivariate ICU physiological time-series forecasting. By simultaneously correcting five persistent methodological deficiencies in the comparative literature (unifying experimental conditions, standardizing preprocessing, introducing VAR as a multivariate linear baseline, reporting variance across 10 runs, and precisely defining the clinical prediction task), this work provides a reliable, reproducible foundation for evidence-based model selection.

LSTM achieves the highest predictive accuracy across all metrics and both datasets, with RMSE 0.082 ± 0.004 and directional accuracy 90.1%±0.9% on MIMIC-III, with all superiority claims statistically validated. GRU offers a compelling efficiency alternative, reaching 97.2% of LSTM's directional accuracy at 64% of training cost. The three-way decomposition enabled by the VAR baseline reveals that gated temporal memory accounts for the largest marginal gain (−47% to −53% RMSE over ANN), followed by nonlinear approximation (−19% over VAR) and multivariate modeling (−13% over ARIMA). Comprehensive ablation studies identify normalization as the single most critical preprocessing step, and *l* = 10 as the optimal sequence length for this clinical task.

Beyond accuracy, this study provides novel clinical evaluations: alarm simulation demonstrates a 38.4% false alarm reduction for LSTM relative to threshold-only monitoring, and subgroup fairness analysis identifies a systematic performance gap for severely ill patients (SOFA ≥ 8) that warrants targeted future research. Cross-hospital generalization experiments confirm that model rankings hold under distributional shift, while revealing that domain adaptation strategies are needed to close the performance gap for high-severity patients and unseen clinical environments.

These results offer actionable guidance for ICU forecasting model selection and establish a reusable benchmark for evaluating next-generation architectures including Transformers, hybrid models, and foundation models in the clinical time-series domain.

## Data Availability

The original contributions presented in the study are included in the article/supplementary material, further inquiries can be directed to the corresponding author/s.
